# Changes in Physical Performance Following Operational Military Training: A Meta-Analysis

**DOI:** 10.1186/s40798-025-00815-y

**Published:** 2025-02-13

**Authors:** Andy Murray, John J. Fraser, David M. Bazett-Jones, Grant E. Norte

**Affiliations:** 1 Department of Exercise and Rehabilitation Sciences, College of Health and Human Services, University of Toledo, Toledo, OH 43606 USA; 2 Department of Physical Medicine and Rehabilitation, School of Medicine, Uniformed Services University of the Health Sciences, Bethesda, MD USA; 3 Sports Medicine Research Institute College of Health Sciences, University of Kentucky, Lexington, KY USA; 4 Department of Health and Human Performance, High Point University, Congdon School of Health Sciences, High Point, NC USA; 5 Cognition, Neuroplasticity, & Sarcopenia (CNS) Lab, Institute of Exercise Physiology and Rehabilitation Science, University of Central Florida, Orlando, FL USA

**Keywords:** Military, Physical performance, Fitness, Operational training, Sports performance

## Abstract

**Background:**

To best simulate armed combat-related emergencies, military personnel undergo operational training that attempts to recreate multiple physical stressors. Understanding the specific aspects of physical performance degradation after operational training helps to better inform future training practices, as these declines translate to real-world operations. The purpose of this meta-analysis was to investigate the effects of operational trainings on physical performance metrics in military personnel.

**Methods:**

Six electronic databases were searched for studies that investigated physical performance metrics in active-duty military personnel before and after multiple-day operational training. Sample sizes, means and standard deviations were extracted from included studies, and random-effects meta-analyses were conducted using standardized mean differences (Hedge’s *g*) with 95% confidence intervals (*p* ≤ 0.05).

**Results:**

Seventeen studies (N = 1592 participants) were included for final review and grouped by physical performance metric. Meta-analyses revealed a large pre-to-post decline in lower body jump power (n = 4, g = 0.87, 95% CI [0.28, 1.47]), and small declines in short-duration lower body power-jump distance (n = 5, g = 0.39, 95% CI [0.16, 0.63]), upper body endurance (n = 4, g = 0.40, 95% CI [0.09, 0.71]), and core endurance (n = 2, g = 0.46, 95% CI [0.10, 0.82]). Substantial heterogeneity was observed in the included studies (*I*^*2*^ = 0–91%).

**Conclusions:**

Military operational training temporarily diminishes lower body performance to a greater extent than other constructs. The most consistent findings showed reductions in power production (large magnitude) and muscular endurance (small magnitude), which appear to have implications for military training and risk reduction. In real-world operational applications, reduced power- and endurance-generating capabilities may be detrimental to meeting tactical requirements, making these metrics valuable for military leadership to focus on during personnel training.

**Supplementary Information:**

The online version contains supplementary material available at 10.1186/s40798-025-00815-y.

## Key Points

- Though varied in duration and exact structure, military operational training can induce physical performance decrements in personnel that undergo such training, including reductions in lower body power, upper body endurance, and core endurance.
- These physical performance declines are thought to be temporary yet reflect the conditions that military personnel are subject to in real operational environments, suggesting that there is a risk of physical performance declines during real military operations.
- Leadership could reduce the impact of these declines by providing ample training opportunities prior to real military operations and by emphasizing the importance of proper recovery when at all possible.

**Supplementary Information:**

The online version contains supplementary material available at 10.1186/s40798-025-00815-y.

## Background

Military operations are uniquely characterized by high physical workloads in hostile environments, often accompanied by fatigue, sleep deprivation, and reduced caloric intake [1, 2]. To best prepare for the level of stress induced by military contingency operations, personnel are required to perform training tasks that simulate combat and/or survival scenarios in various environments [3, 4]. Performing ecologically valid and highly variable tasks, including sustained physical activity in a sleep-deprived state [5], can elicit a great degree of mental, psychological, and physical stress [6] similar to that experienced during armed combat or emergency response [5]. The philosophy of “train as you fight” and realism during training is highly valued by the military to best prepare service members for wide-spectrum challenges experienced during expeditionary operations [7].

Combat endurance is another salient factor during military training, as sustained operations typically involve persistent physical stressors without adequate recovery periods [6]. While operational training frequently involves variable mission tasks (e.g. survival training tasks may differ from combat simulation), the multi-stressor elements of sleep deprivation, near-constant physical activity, low caloric intake, task complexity, and hostile environments are typically consistent in both training and real-world operations. The duration of intensive military training varies widely, frequently ranging from 24 h [8] to multiple weeks [9]. To emulate the intensity of sustained operations, training is often designed to occur consecutively with little rest between events. For example, candidates enrolled in the US Army Ranger Training incur cumulative stress during a 62-day multiple simulative exercise [3], with the objective of improving occupational skills [3], physical conditioning [3], stress tolerance [10], and resiliency [10] needed during highly dynamic and hazardous kinetic operations. Multi-stressor operational trainings are reported to negatively impact physiological performance, triggering stress-induced endocrine fluctuations and inflammatory biomarkers [11–14]. These declines in physiological function during training are thought to hinder physical performance.

Numerous studies have examined the fitness-related changes that occur during and immediately following training [8, 12, 15–30]. Only one meta-analysis has been conducted on physical performance changes immediately after military training [12], emphasizing assessments of strength and power measures and excluding analyses of other fitness constructs, such as muscular endurance and aerobic capacity. In addition to strength and power measures, it is important to understand which metrics of physical performance degrade most during and after operational trainings to best inform training practices. As operational training is designed to emulate real-world operational stressors, the ability to maintain performance during trainings may translate to enhanced operations.

Therefore, the primary purpose of this meta-analysis was to investigate the impact of operational military training on measures of physical performance. We hypothesized that measures of physical performance—upper and lower body power; upper, lower, and full body strength; upper body and core endurance; aerobic endurance; anaerobic power and capacity; and agility-– would decline immediately following training.

## Methods

### Registration

This systematic review was performed following the guidelines detailed by the Preferred Reporting Items for Systematic Reviews and Meta-Analysis (PRISMA) statement [31]. The protocol for this review was registered with PROSPERO before the initial search began (registration No: CRD42022287916, registration date: 17 Jan 2022) with no deviations from the registered PROSPERO protocol.

### Eligibility Criteria

The included studies met the following criteria: (a) the study population included active-duty military personnel of any nationality; (b) the intervention studied involved operational training conducted without the use of ergogenic aids; and (c) outcomes of physical fitness performance were assessed before and after training. Studies were excluded if the following characteristics were found: (a) the study included veterans, reserves, pre-military trainees, or non-military populations; or (b) operational training was conducted as a part of a recruit training program instead of as a fully inducted personnel training (e.g. basic training). In the case of studies that did not provide the necessary information, the original authors were contacted. If data were unavailable, studies were excluded.

Operational trainings have frequently been referred to by a variety of different terms, including “sustained operations” [7, 32], “military environmental survival simulation training” [15], and “simulated military operational stress” [33]. In order to best represent the multitude of differently named trainings that focus on similar goals, program designs that met our inclusion criteria were collectively referred to as “operational training”.

### Information Sources and Search Strategy

The initial search was conducted using the electronic databases of PubMed, ProQuest, Defence Technical Information Center (DTIC), Embase, CINAHL, Web of Science, and SPORTDiscus from inception to 05 July 2022. The search strategy was developed through an iterative process in consultation with a university research librarian, and included terms that specified population (e.g. military, soldiers, army, navy, etc.), trainings (e.g. operational training, sustained operations training, simulated operational training, field training exercise, etc.), and outcomes of interest (e.g. physical fitness, physical activity, etc.). Search terms were modified as necessary for each database (Appendix 1). The search was limited to studies available in the English language. Grey literature (e.g. military technical reports) were not included in this review. Study designs that were considered for this review included case–control studies, observational studies, and experimental trials with a control group, including randomized controlled trials. In experimental trials, only participants from the control arm were included to account for any potential confounding variables introduced to the experimental arm.

### Study Selection

**Fig. 1:**
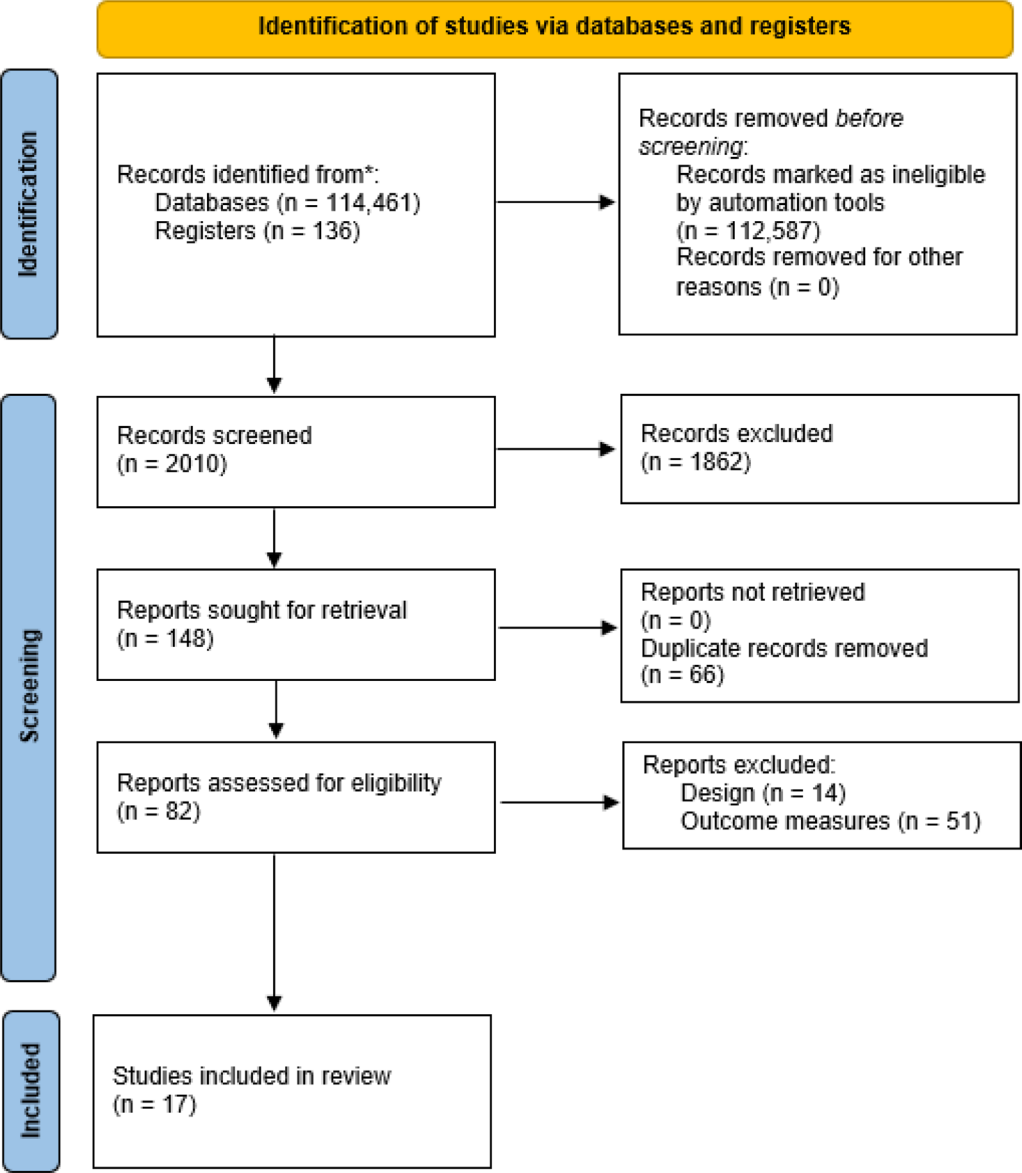
PRISMA Flow Diagram

One investigator (AAM) exported all studies meeting initial search criteria to Endnote 20.2.1 (Clarivate Analytics) and removed duplicates (Fig. 1). One investigator (AAM) independently screened titles and abstracts for inclusion; studies not clearly meeting criteria were discussed with a second reviewer (GEN**)** prior to exclusion. After excluding studies based on title and abstract, each remaining full-text study was assessed and evaluated based on the full eligibility criteria.

### Data Extraction

Two reviewers (AAM, GEN) independently extracted the following data from each included study: (a) publication information, (b) participant demographics, including sample size, age, sex, years of service, prior injury status, and baseline fitness status as available, (c) study methods, including training design (types and degree of training-related stressors, days of operational training, participant dropout, and any comments on recovery period testing for the few studies that included that component), fitness assessments and additional methodological considerations, (d) type of data analysis performed, and (e) relevant outcome measures, including indicators of physical fitness and any within-group differences pre-intervention to post-intervention concluded. Any conflicts were resolved through dialogue between the reviewers.

### Outcome Measures

The outcome measures included nine constructs of physical fitness, with four being further subcategorized according to the similarity of activity performed using the National Strength and Conditioning Association exercise prescription guidelines [34]. Constructs were established after evaluation of all included studies and the physical fitness measures conducted within the included studies. The constructs and their basic definitions are as follows:

- Upper Body Power—activities involving the use of the upper extremities through high-intensity, high-velocity movement (e.g., a weighted ball throw for distance).
- Lower Body Power—activities involving the use of the lower extremities through high-intensity, high-velocity movement. This construct was subdivided into a “Jump Distance” subcategory (e.g., vertical or broad jump distance) and a “Jump Power” subcategory (e.g., maximal exertion vertical jumps power).
- Upper Body Strength—activities involving the use of the upper extremities through high-intensity, low-velocity movement. This construct was subdivided into a “Handgrip” subcategory, (e.g., handgrip strength via handheld dynamometer), and an “Other” subcategory (e.g., maximal bench press weight).
- Lower Body Strength—activities involving the use of the lower extremities through high-intensity, low-velocity movement (e.g., leg extension force).
- Full Body Strength—activities involving the use of both the upper and lower extremities through high-intensity, low-velocity movement (e.g., lifting of a weight from ground to chest-level).
- Muscular Endurance—activities involving the use of low-intensity, high-repetition movement. This construct was subdivided into an “Upper” subcategory (e.g., pushups repetitions in a given time) and a “Core” subcategory (e.g., sit-ups repetitions in a given time).
- Aerobic—activities involving the use of low-medium-intensity, long-duration cardiovascular movement (e.g., 4 km run) or the use of standard aerobic assessments (e.g., maximal oxygen uptake (VO_2_max) Test).
- Anaerobic—activities involving the use of high-intensity, short-duration cardiovascular movement. This category was subdivided into a “Capacity” subcategory (e.g., 300-yard run) and a “Power” subcategory (e.g., 20 m sprint).
- Agility—activities involving both speed and coordination movements (e.g. agility run drill).

### Methodological Quality Assessment

The methodological quality of all included studies was independently assessed by two reviewers (AAM, GEN) using a modified version of the National Institutes of Health (NIH) Quality Assessment Tool for Before-After (Pre-Post) Studies with No Control Group (Appendix 2). The original NIH Quality Assessment Tool for Before-After (Pre-Post) Studies with No Control Group scale consists of 12 questions that evaluate qualitative methodological value. Questions that were not applicable to the results of the selected studies were eliminated (Question #4, 8, and 11). Thus, a total of 9 points were assignable through the modified scale. The criteria included in this scale were discussed and agreed upon by each reviewer prior to conducting the quality assessment. Any differences in ratings between the two reviewers were discussed and resolved. The median score of all included studies was calculated. Studies were categorized as low-quality (< median), moderate quality (equal to the median), and high-quality (> median).

### Statistical Methods

Statistical analysis was performed with Review Manager 5 (The Cochrane Collaboration, Copenhagen, Denmark). Initial analyses were conducted by the primary reviewer (AAM) and confirmed by a second investigator (GEN). Standardized mean differences (SMD) with 95% confidence intervals (CI) were calculated, described with Hedge’s *g* effect sizes, and displayed on forest plots. SMD were categorized as small (≤ 0.50), medium (0.51–0.79), or large (≥ 0.80). Separate random-effects meta-analyses were performed to determine the magnitude of within-group change in performance outcomes following training. Percent change pre-post intervention with CI values were calculated for outcomes with significant findings. Subgroup comparisons were included for studies based on the visual inflection point of intervention duration distribution; “short” interventions were assigned based on a duration of less than three weeks, and “long” interventions were assigned based on a duration equal to or greater than three weeks. For similar methods and outcome measures between at least two included studies, a meta-analysis was conducted. Risk of bias across studies was evaluated via funnel plots with Egger’s test using StatsDirect (StatsDirect Ltd., Merseyside, UK) for categories with significant results and with at least three studies, as per the Cochrane Handbook recommendations for testing funnel plot asymmetry [35]. The statistical heterogeneity level for pooled data was created using the *I*^2^ statistics (heterogeneity = *I*^2^ > 50%) [35].

### Levels of Evidence

The level of evidence for each construct and, if applicable, subcategory included in this meta-analysis were assessed using the Van Tulder criteria [36] for observational design studies (Appendix 3). Levels of evidence were categorized as strong, moderate, limited, or very limited based on the heterogeneity, quality of the included studies, and quantity of high-quality studies in each construct. Strong evidence was classified as including statistically homogeneous pooled results from three or more studies, including a minimum of two high-quality studies. Moderate evidence was classified as including statistically heterogeneous pooled results from multiple studies, including at least one high-quality study, or from multiple statistically homogeneous moderate-quality or low-quality studies. Limited evidence was classified as including statistically heterogeneous pooled results from multiple moderate-quality or low-quality studies, or results from one high-quality study during sub-group analyses. Very limited evidence was classified as including results from a single moderate-quality or low-quality study during sub-analyses. Although a 50% threshold was used to determine levels of evidence, the small number of samples for some of the analyses may have affected the validity of this heterogeneity estimate [35].

## Results

### Search Strategy

Full details of search results are available in the PRISMA Flow Diagram (Fig. 1). Upon title and abstract screening, a total of 82 publications were reviewed in full text, of which 17 were included in the final selection of articles. Data from 469 participants were included, with results grouped across the 9 physical fitness constructs. To reduce duplication of the same participant data across multiple outcome measures within one activity construct, the outcome measure that best aligned with the construct was included. The participant demographics for each included study are provided in Table 1.

**Table 1:** Participant demographics

Study	Participants (#)	Sex	Age (years)	Type of training	Training duration
Chester et al. [1]	14	9 male 5 female	28.0 ± 8.8	Environmental survival training	15 days
Gan et al. [2]	30	30 male	24.3 ± 3.1	Singapore Ranger course	62 days
Hoffman et al. [3]	10	10 male	20.2 ± 1.1	Sustained military operations	28 days
Margolis et al. [4]	21	21 male	20.0 ± 1.0	Military task training with ski march	7 days
Montain et al. [5]	10	10 male	30.0 ± 4.0	Army Ranger field training	6 days
Nindl et al. [6]	10	10 male	22.0 ± 3.0	Sustained operations	4 days
Nindl et al. [7]	50	50 male	24.6 ± 4.4	US Army Ranger training	62 days
Ojanen et al. [8]	49	49 male	20.0 ± 1	Prolonged military field training	21 days
Ojanen et al. [9]	49	49 male	20.0 ± 1	Prolonged military field training	21 days
Ritland et al. [10]	54	54 male	25.1 ± 4.0	Night-time Operations	1 day
Rozanski et al. [11]	15	15 male	33.1 ± 3.5	Survival training	2 days
Salonen et al. [12]	10	10 male	20.0 ± 1	Field training	7 days
Sporis et al. [13]	12	Not Reported	24.4 ± 2.6	Croatian Special Forces training	62 days
Szivak et al. [14]	20	20 male	25.3 ± 3.6	US Navy Survival, Evasion, Resistance, and Escape (SERE) training	10 days
Tomczak et al. [15]	13	13 male	36.3 ± 8.3	Survival training	1.5 days
Welsh et al. [16]	29	Not Reported	24.0 ± 1.0	Sustained operations	8 days
Winters et al. [17]	73	Not Reported	27.4 ± 3.8	Marine Raider Individual Training Course	9 months

### Methodological Quality Assessment

The median score for methodological quality was six out of nine, with scores ranging from five to eight (Electronic Supplementary Material Figure S1). Of the seventeen included studies, six (35.3%) were rated as above-average quality, nine (52.9%) as average quality, and two (11.8%) as below-average quality. When assigning a level of evidence for each construct, methodological quality was clarified using the following: above-average as “high”, average as “moderate”, and below-average as “low” quality. Individual assessment for each included study is provided in the electronic supplementary material (Electronic Supplementary Material Figure S2).

### Risk of Publication Bias

Egger’s regression test and funnel plots were used to evaluate the effect size of significant findings on the risk of publication bias. Of all the evaluated constructs or subcategories, only four were statistically different following sustained operations: Lower Body Power: Jump Distance, Lower Body Power: Jump Power, Muscular Endurance: Upper Body Endurance, and Muscular Endurance: Core Endurance. Since Egger’s test requires at least 10 studies to be powered appropriately [35], only Lower Body Power: Jump Distance was able to be evaluated using this method. Funnel plots with Egger’s test are provided in the electronic supplementary material (Electronic Supplementary Material Figures S2-S4). No risks of bias were found for Lower Body Power: Jump Distance (intercept =  − 0.56, *p* = .87), Lower Body Power: Jump Power (intercept = 6.05, *p* = .22) or Muscular Endurance: Upper Body Endurance (intercept = 1.01, *p* = .55).

### Upper Body Power

**Fig. 2:**
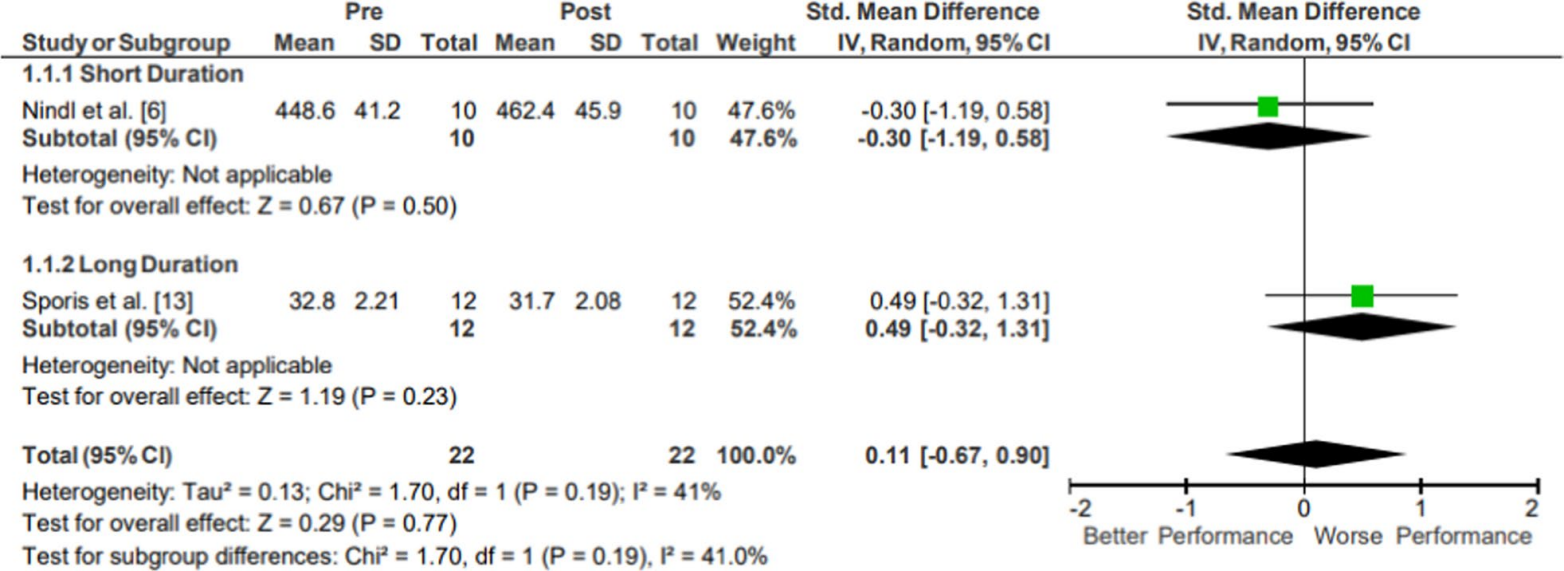
Impact of operational military training on Upper Body Power. Abbreviations: *SD* standard deviation, *CI* confidence interval, *IV* inverse variance

Results of the meta-analysis of Upper Body Power are presented in Fig. 2. Moderate evidence from two mixed-quality studies indicated no significant changes in upper body power following operational training (*p* = .77; *g* = 0.11, 95% CI [− 0.67, − 0.90]) [20, 26]. One study was classified as a short duration intervention [20] and the other as a long duration intervention [26], with a total of 22 participants. The movements examined differed in muscular involvement and in positioning; the short duration study evaluated power from bench press throws [20], while the long duration study evaluated medicine ball throw distance [26].

### Lower Body Power

#### Jump Distance

**Fig. 3:**
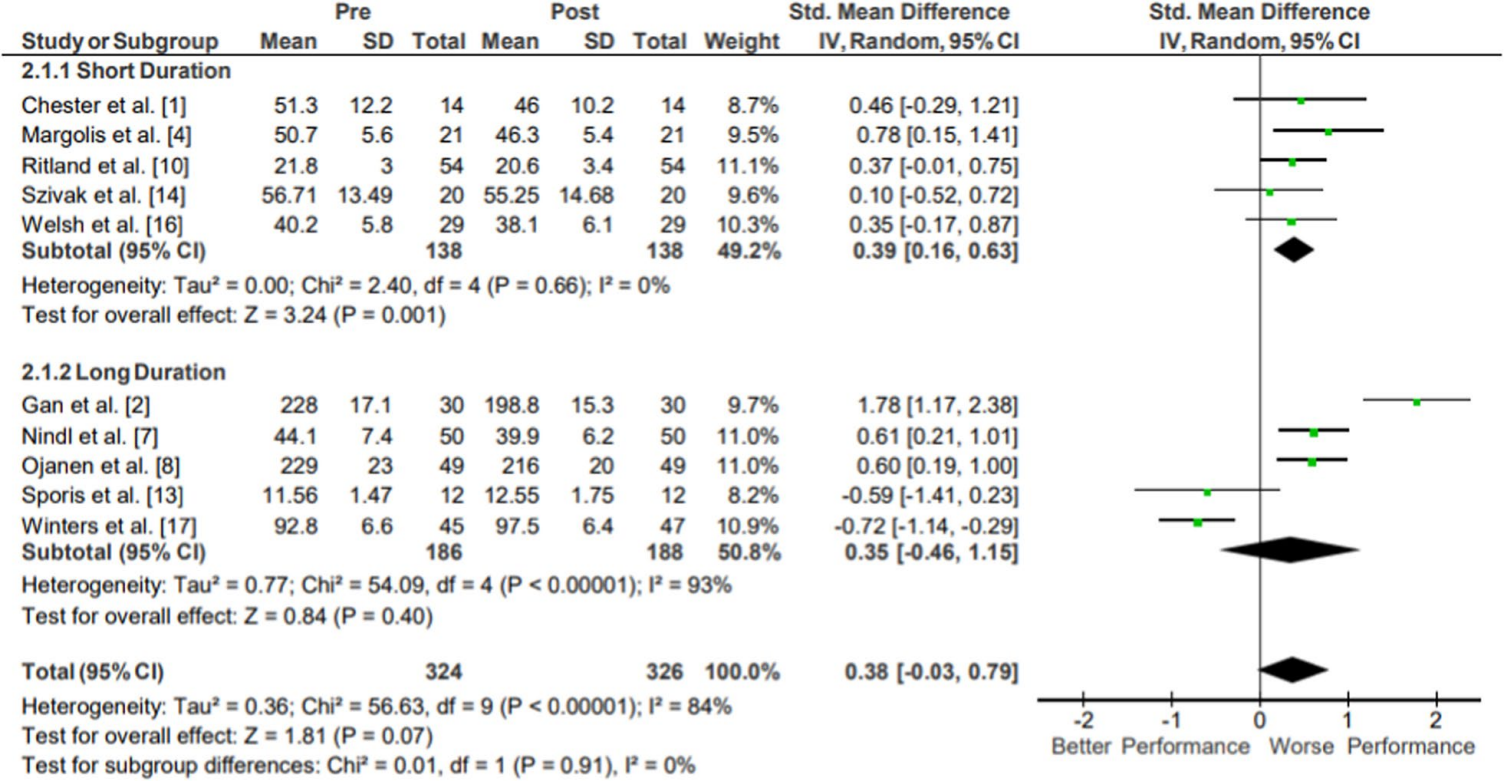
Impact of operational military training on Lower Body Power: Jump Distance. Abbreviations: *SD* standard deviation, *CI* confidence interval, *IV* inverse variance

Results of the meta-analysis of Lower Body Power: Jump Distance are presented in Fig. 3. Strong evidence from five mixed-quality short-duration studies showed a small magnitude reduction in jump distance (*g* = 0.39, 95% CI [0.16, 0.63]; 6.55% decrease, 95% CI [5.48, 7.62]) with significant overall effect (*p* < .001) and low heterogeneity (*I*^2^ = 0%) [15, 18, 23, 27, 29]. Moderate evidence from five mixed-quality long-duration studies indicated no significant changes in jump distance following operational training [8, 16, 21, 26, 30]. Moderate evidence jump distance pooled data from 324 participants indicated no significant changes in jump distance following operational training. The movements performed in all included studies examined either vertical or horizontal jumping distances, with the horizontal tests including either standing broad jumps or long jumps.

#### Jump Power

**Fig. 4:**
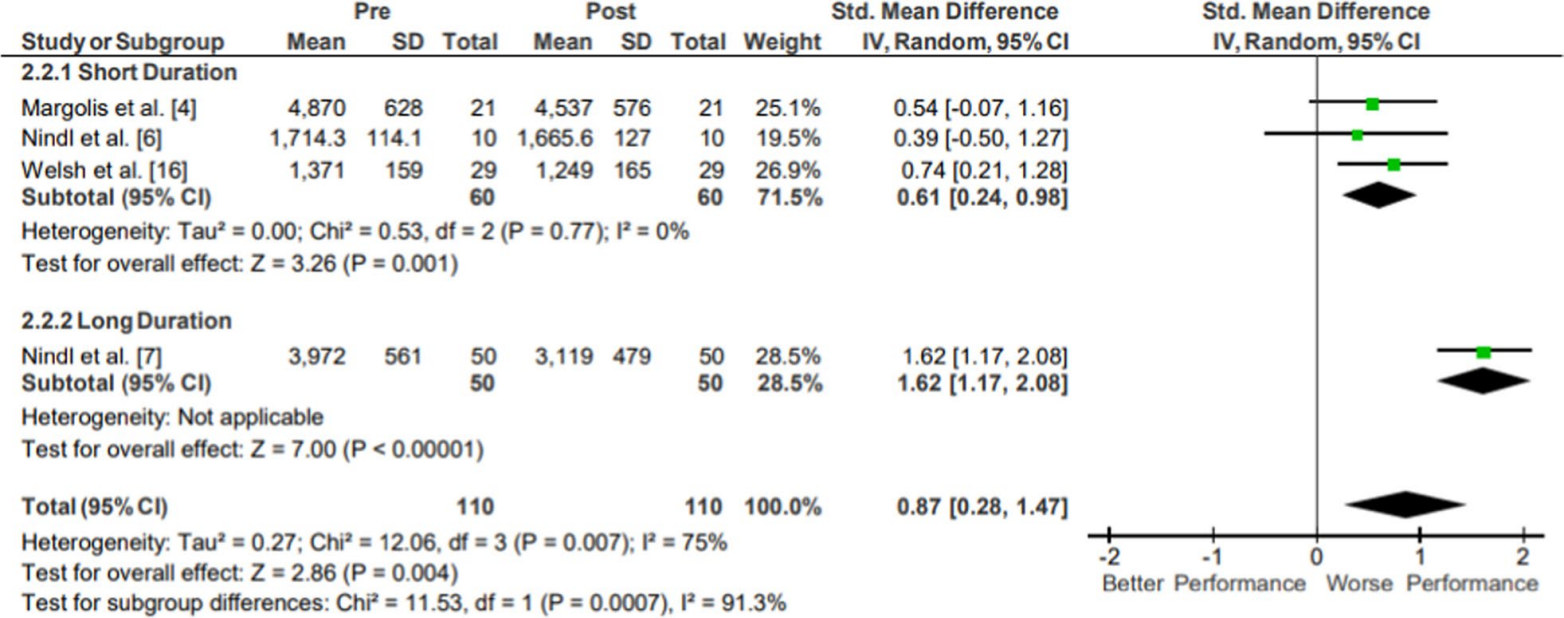
Impact of operational military training on Lower Body Power: Jump Power. Abbreviations: *SD* standard deviation, *CI* confidence interval, *IV* inverse variance

Results of the meta-analysis of Lower Body Power: Jump Power are presented in Fig. 4. Moderate evidence from three mixed-quality short-duration studies showed a medium magnitude reduction in jump power (*g* = 0.61, 95% CI [0.24, 0.98]) with significant overall effect (*p* < .001) and low heterogeneity (*I*^2^ = 0%) [18, 20, 29]. Limited evidence from one low-quality long duration study could not be quantitatively evaluated due to too small of a sample size (N = 1) [21]. Limited evidence jump power pooled data from 110 participants showed a significant and large magnitude reduction in jump power (*p* = .004; *g* = 0.87, 95% CI [0.28, 1.47]; 11.37% decrease, 95% CI [− 28.37, 51.12]; *I*^2^ = 91.3%). The movements performed in all included studies examined peak vertical jumping power, with one specifically evaluating squat jump power [20].

### Upper Body Strength

#### Handgrip

**Fig. 5:**
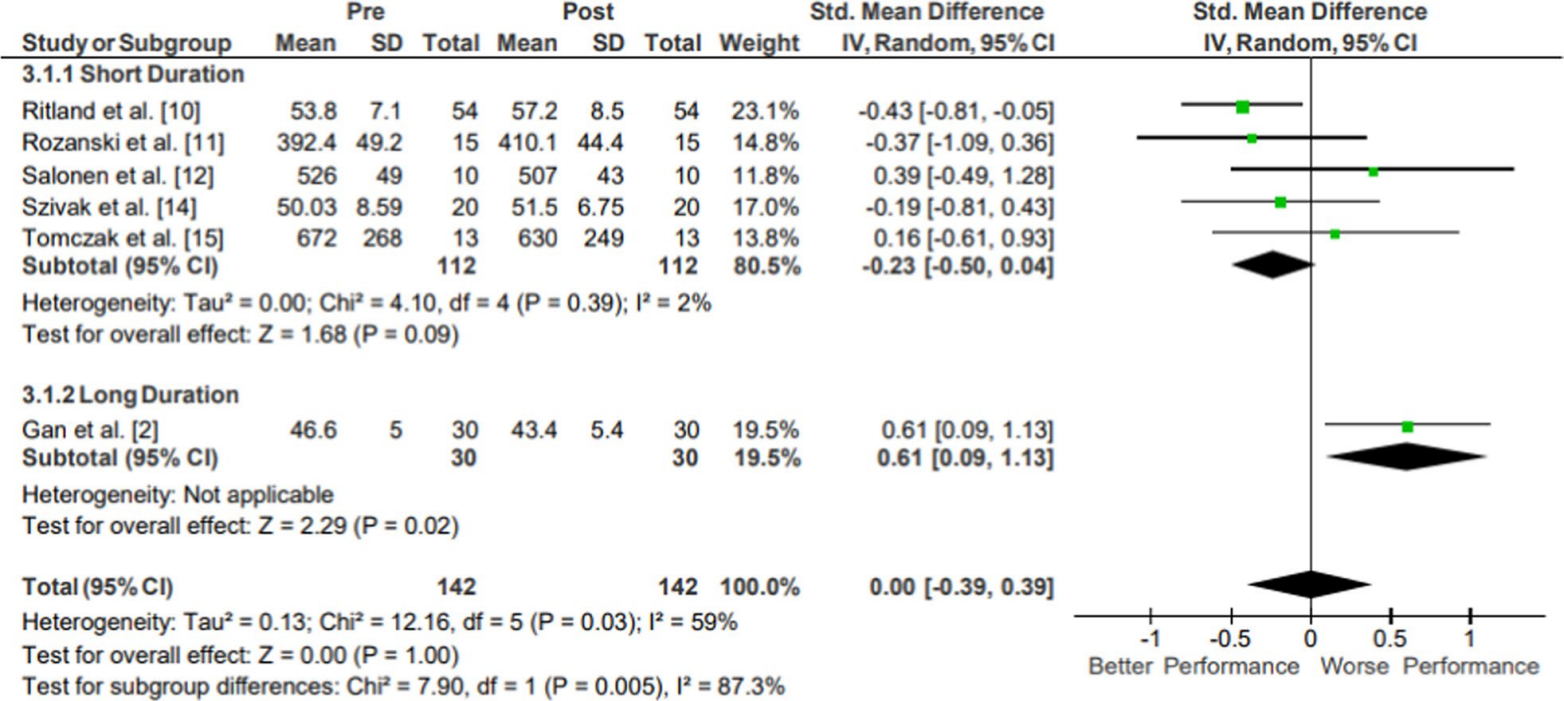
Impact of operational military training on Upper Body Strength: Handgrip. Abbreviations: *SD* standard deviation, *CI* confidence interval, *IV* inverse variance

Results of the meta-analysis of Upper Body Strength: Handgrip are presented in Fig. 5. Moderate evidence from five mixed-quality short-duration studies showed no significant changes in handgrip strength following operational training (*p* = 1.00; *g* = 0.00, 95% CI [− 0.39, 0.39]) [23–25, 27, 28]. Moderate evidence from one high-quality long-duration study could not be evaluated due to too small of a sample size (N = 1) [16]. Moderate evidence handgrip strength pooled data from 142 participants indicated no significant changes following operational training. All included studies examined handgrip strength using a handgrip dynamometer.

#### Other

**Fig. 6:**
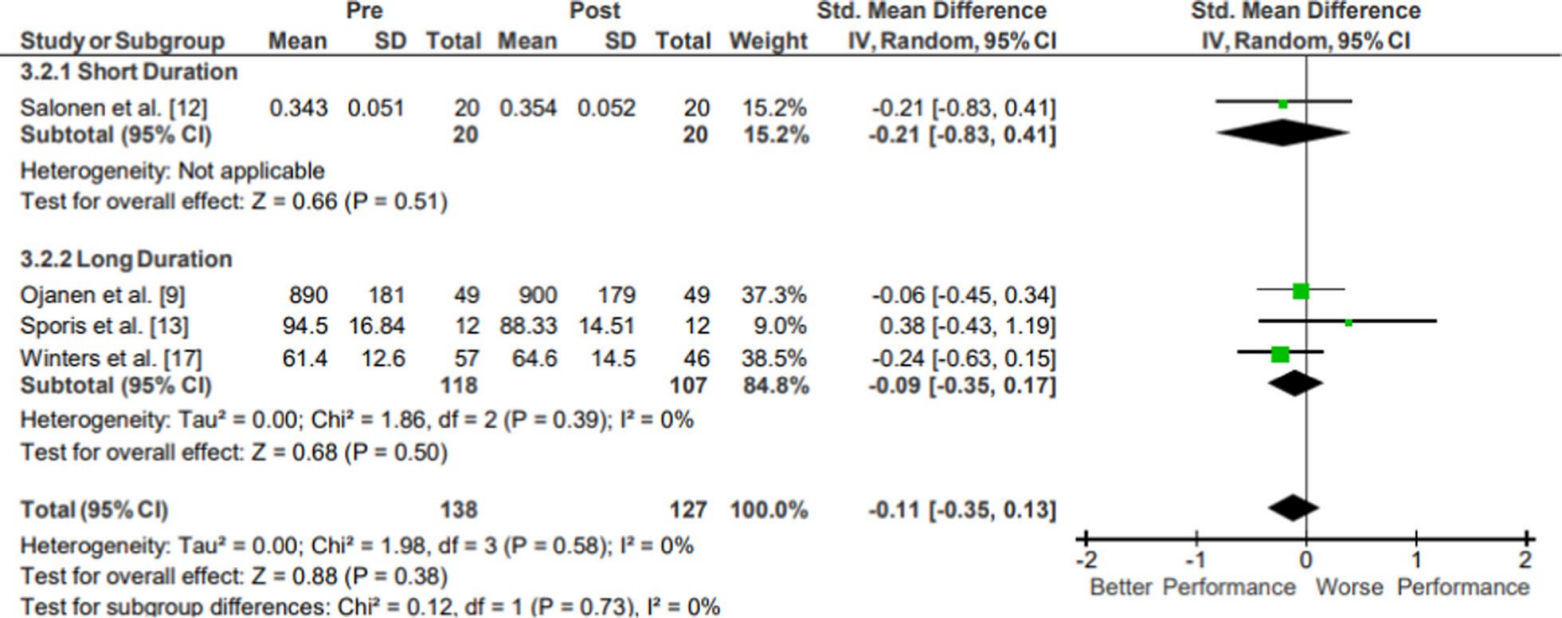
Impact of operational military training on Upper Body Strength: Other. Abbreviations: *SD* standard deviation, *CI* confidence interval, *IV* inverse variance

Results of the meta-analysis of Upper Body Strength: Other are presented in Fig. 6. Strong evidence from four mixed-quality long-duration studies showed no significant changes in upper body strength following operational training (*p* = .38; *g* =  − 0.11, 95% CI [− 0.35, 0.13]) [22, 26, 30]. Moderate evidence from one low-quality short-duration study could not be evaluated due to too small of a sample size (N = 1) [25]. Strong evidence upper body strength pooled data from 113 participants indicated no significant changes following operational training. The movements from included studies varied, evaluating bench press force, arm flexion force, and internal shoulder rotational force.

### Lower Body Strength

**Fig. 7:**
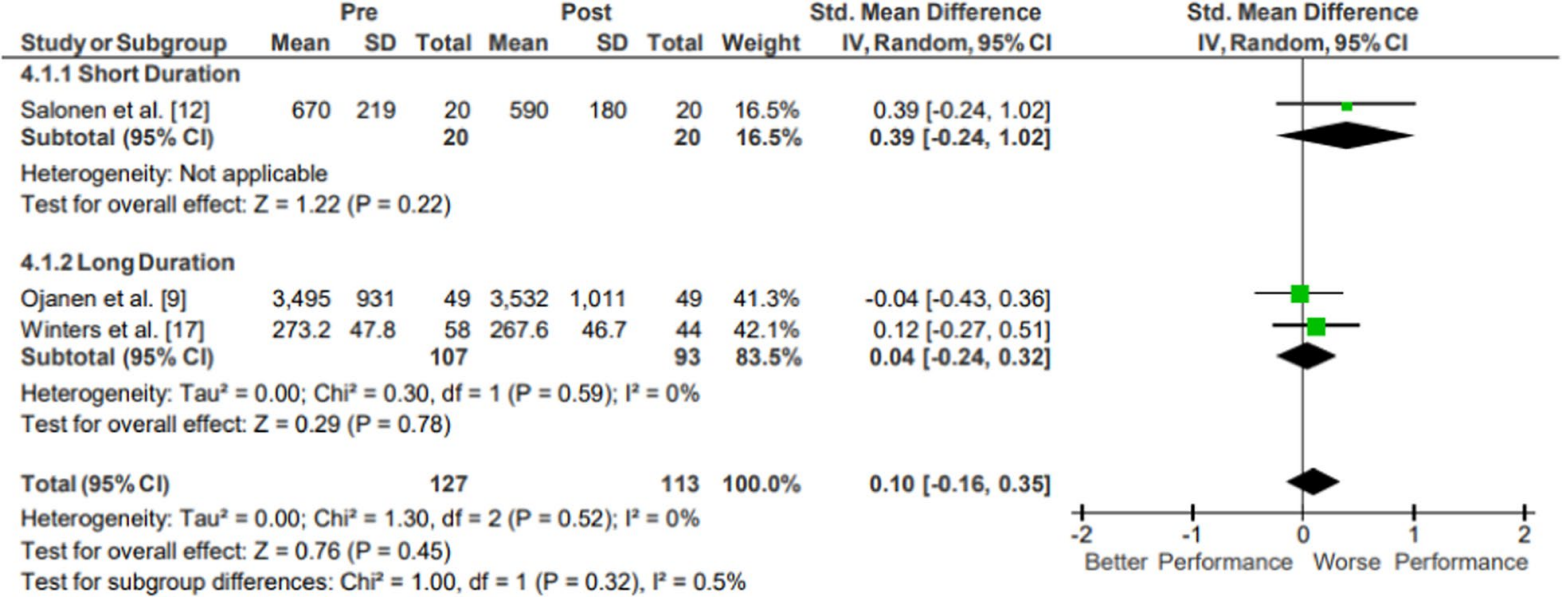
Impact of operational military training on Lower Body Strength. Abbreviations: *SD* standard deviation, *CI* confidence interval, *IV* inverse variance

Results of the meta-analysis of Lower Body Strength are presented in Fig. 7. Moderate evidence from two mixed-quality long-duration studies showed no significant changes in lower body strength following operational training (*p* = .45; *g* = 0.10, 95% CI [− 0.16, 0.35]) [22, 30]. Moderate evidence from one low-quality short-duration study could not be evaluated due to too small of a sample size (N = 1) [25]. Moderate evidence lower body strength pooled data from 127 participants indicated no significant changes following operational training. The movements from the included studies varied, evaluating leg extension force and leg press force.

### Full Body Strength

**Fig. 8:**
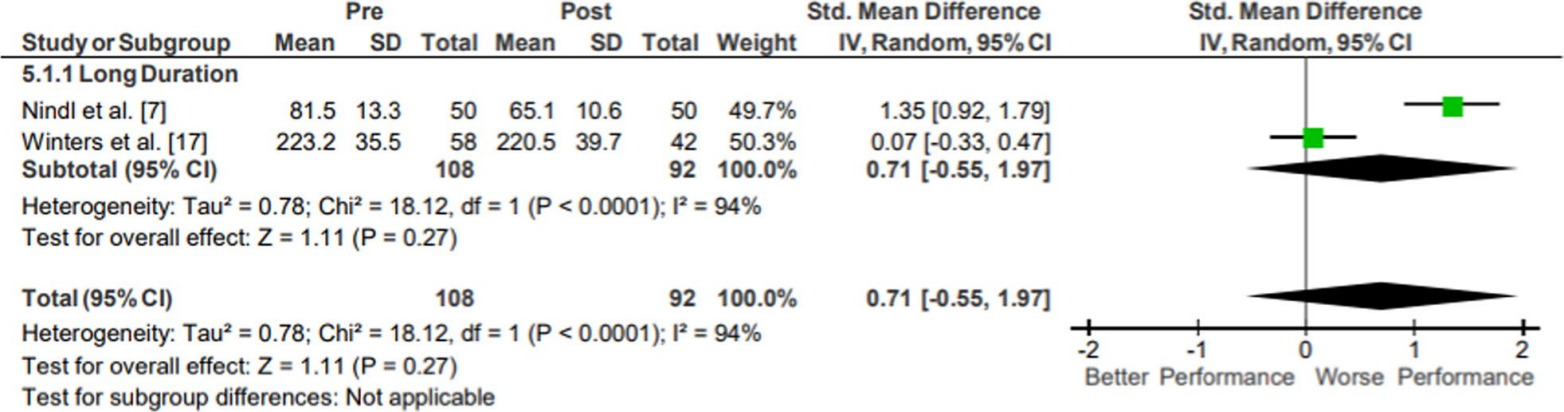
Impact of operational military training on Full Body Strength. Abbreviations: *SD* standard deviation, *CI* confidence interval, *IV* inverse variance

Results of the meta-analysis of Full Body Strength are presented in Fig. 8. Moderate evidence from two mixed-quality studies showed no significant changes in full body strength following operational training (*p* = .27; *g* = 0.71, 95% CI [−0.55, 1.97]) [21, 30]. Both studies were classified as long duration interventions, with a total of 92 participants. The movements from the included studies varied, evaluating trunk flexion force and strength from a power clean-type motion.

### Muscular Endurance

#### Upper Body Endurance

**Fig. 9:**
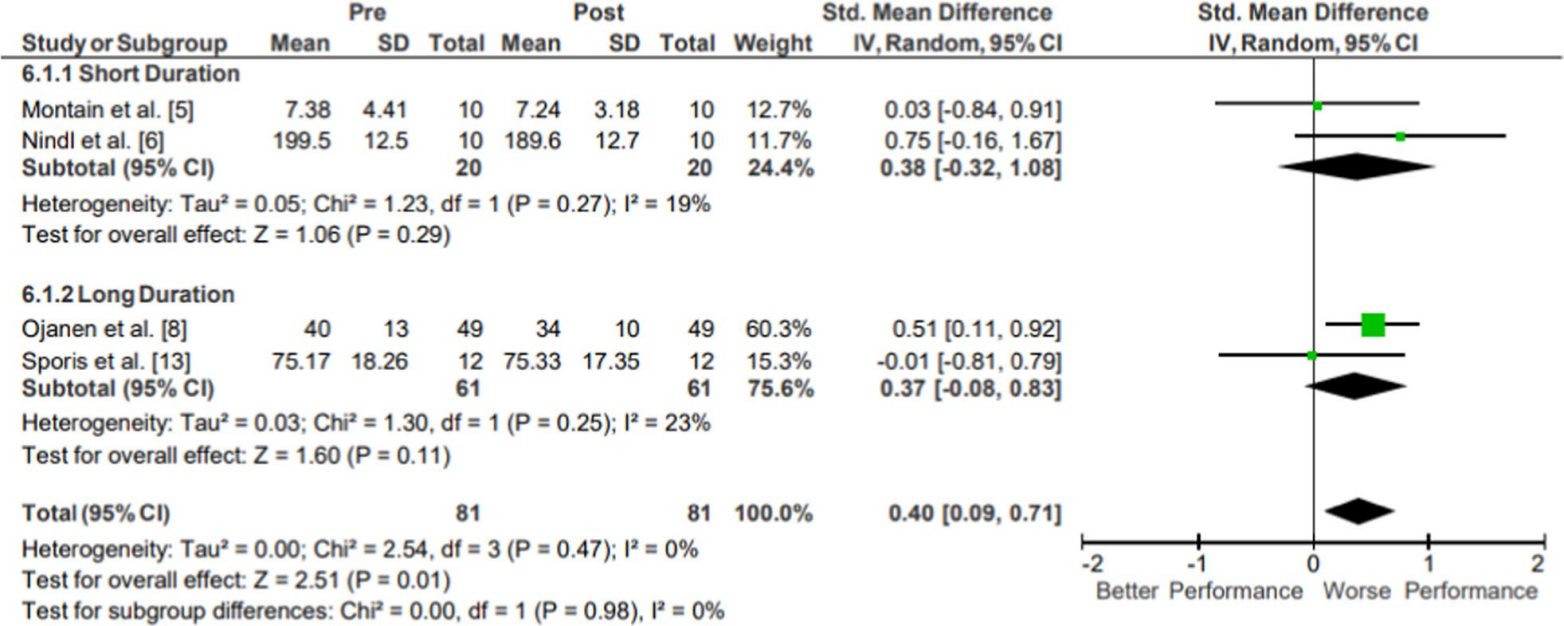
Impact of operational military training on Muscular Endurance: Upper Body Endurance. Abbreviations: *SD* standard deviation, *CI* confidence interval, *IV* inverse variance

Results of the meta-analysis of Muscular Endurance: Upper Body Endurance are presented in Fig. 9. Moderate evidence from two moderate-quality short-duration studies showed no significant changes in upper body endurance following operational training [19, 20]. Moderate evidence from two moderate-quality long-duration studies showed no significant changes in upper body endurance following operational training [8, 26]. Moderate evidence upper body endurance pooled data from 81 participants showed a significant and small magnitude reduction in upper body endurance (*p* = .01; *g* = 0.40, 95% CI [0.09, 0.71]; 4.93% decrease, 95% CI [3.78, 6.08]; *I*^2^ = 0%). The movements from the included studies varied, assessing rock climb time [19], box lift reps [20], and pushup count [8, 26].

#### Core Endurance

**Fig. 10:**
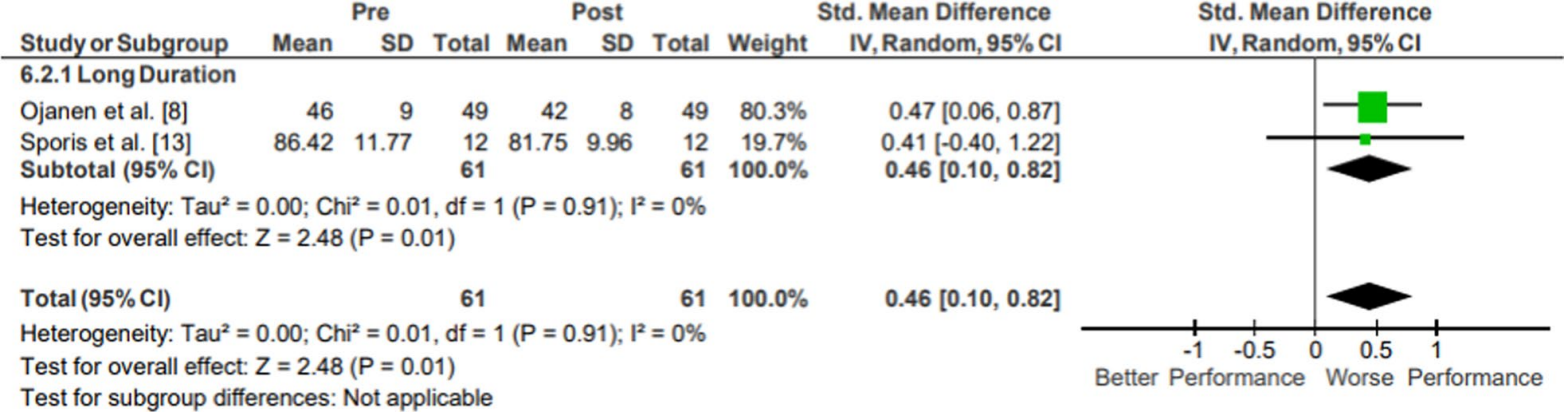
Impact of operational military training on Muscular Endurance: Core Endurance. Abbreviations: *SD* standard deviation, *CI* confidence interval, *IV* inverse variance

Results of the meta-analysis of Muscular Endurance: Core Endurance are presented in Fig. 10. Moderate evidence core endurance pooled data from 61 participants showed a significant and small magnitude reduction in upper body endurance (*p* = .01;* g* = 0.46, 95% CI [0.10, 0.82]; 6.55% decrease, 95% CI [6.14, 6.95]; *I*^2^ = 0%) [8, 26]. Both studies were classified as long duration and assessed to be of moderate methodological quality. Both studies measured sit-up count.

### Aerobic Endurance

**Fig. 11:**
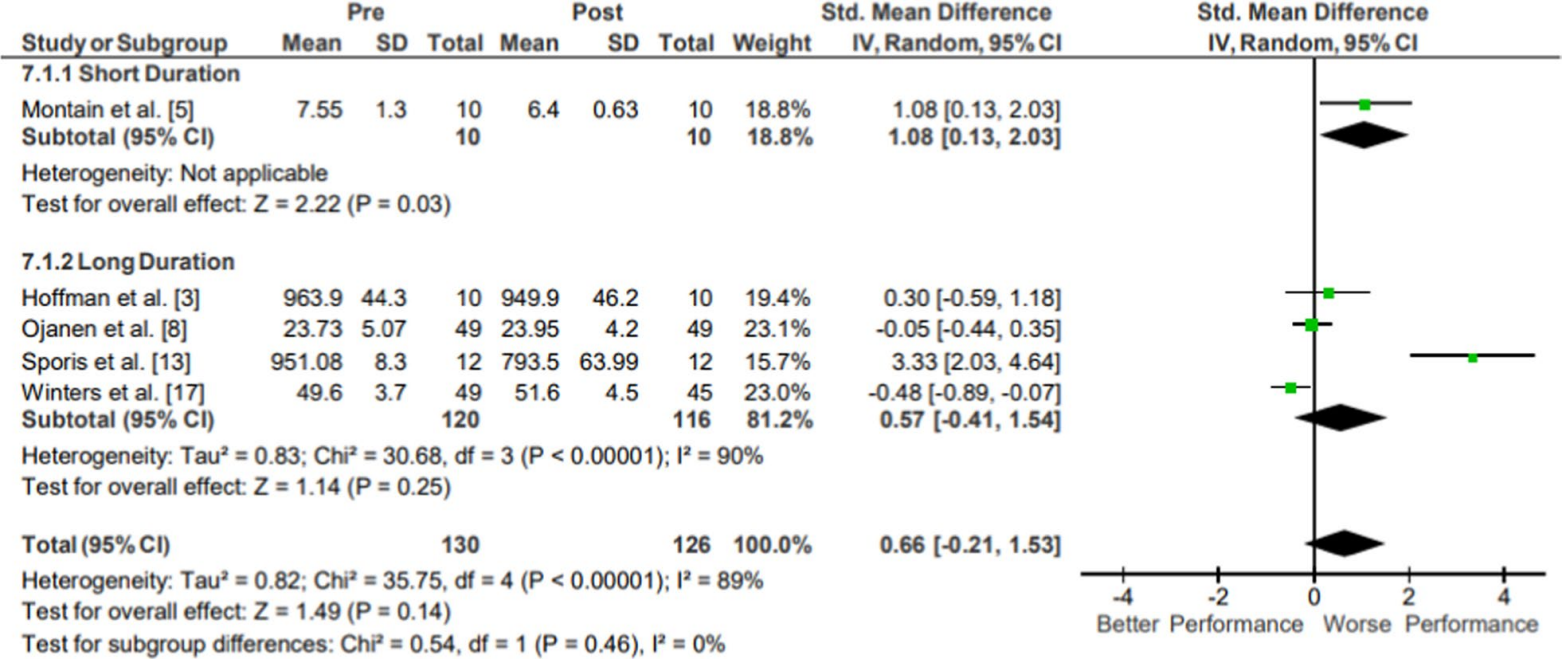
Impact of operational military training on Aerobic Endurance. Abbreviations: *SD* standard deviation, *CI* confidence interval, *IV* inverse variance

Results of the meta-analysis of Aerobic Endurance are presented in Fig. 11. Moderate evidence from four mixed-quality long-duration studies showed no significant changes in aerobic endurance following operational training (*p* = .14; *g* = 0.66, 95% CI [− 0.21, 1.53]) [8, 17, 26, 30]. Moderate evidence from the moderate quality short duration study could not be evaluated due to too small of a sample size (N = 1) [19]. Moderate evidence aerobic endurance pooled data from 126 participants indicated no significant changes following operational training. The movements from the included studies varied, assessing 4 km run time, foot race time, loaded 3.2 km march, 3200 m run time, and VO_2_max.

### Anaerobic

#### Anaerobic Power

**Fig. 12:**
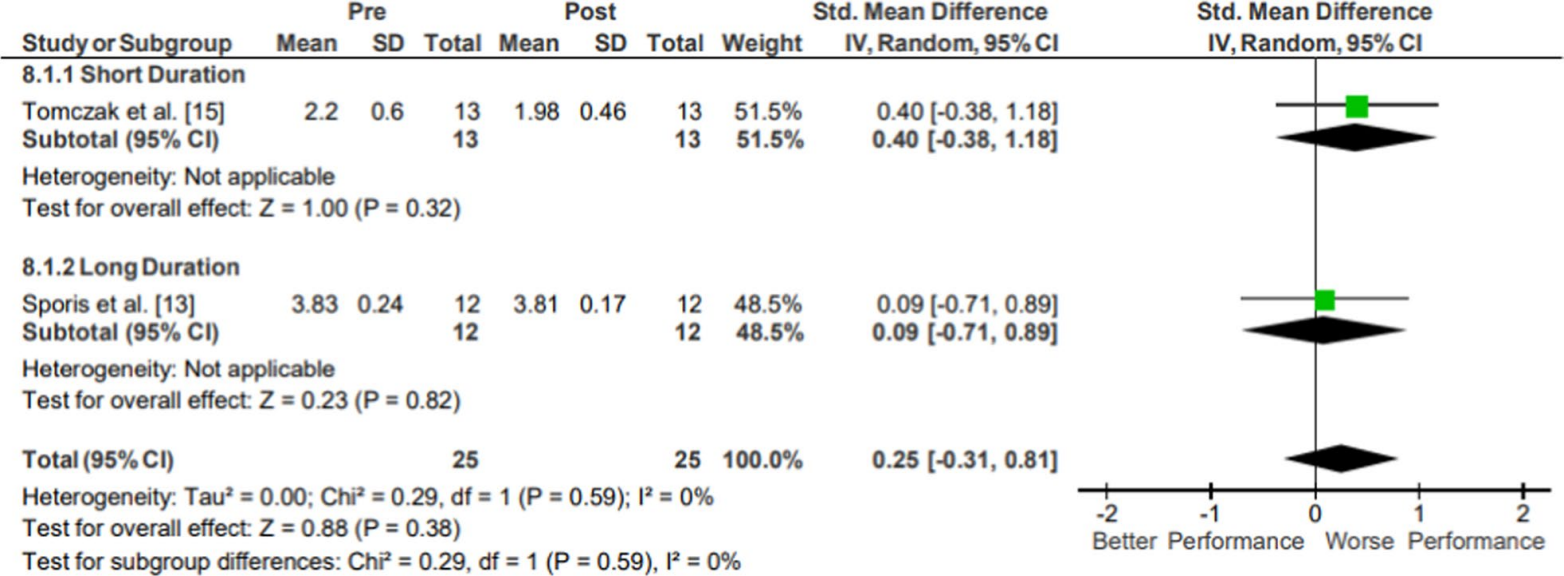
Impact of operational military training on Anaerobic: Anaerobic Power. Abbreviations: *SD* standard deviation, *CI* confidence interval, *IV* inverse variance

Results of the meta-analysis of Anaerobic: Anaerobic Power are presented in Fig. 12. Moderate evidence mixed-quality anaerobic power pooled data from 57 participants indicated no significant changes following operational training (*p* = 0.20; *g* = 0.43, 95% CI [−0.23, 1.08]) [26, 28]. The included studies both assessed sprint times for distances at or under 20 m.

#### Anaerobic Capacity

**Fig. 13:**
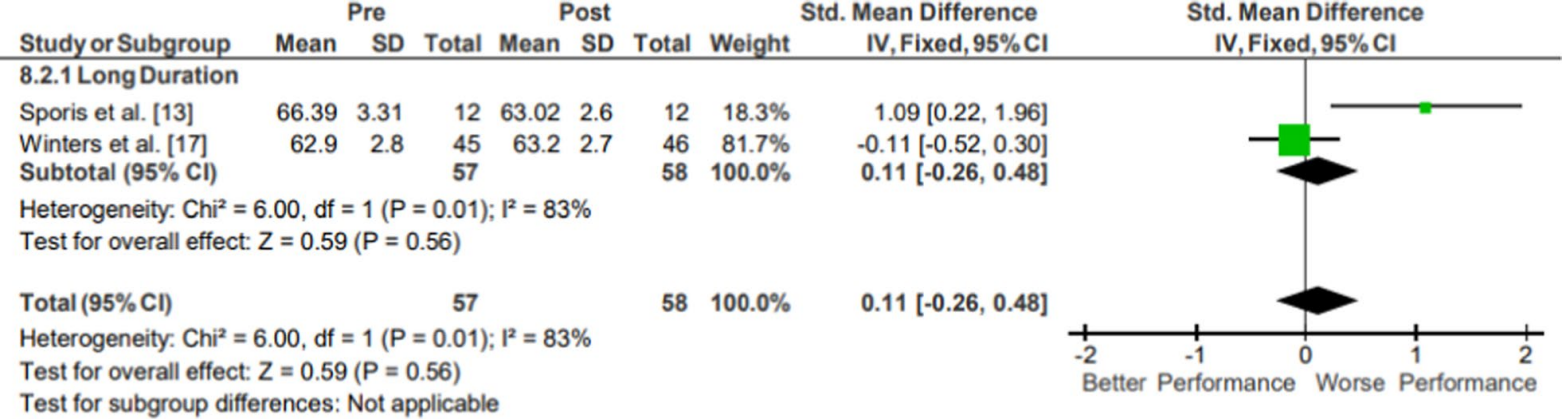
Impact of operational military training on Anaerobic Capacity. Abbreviations: *SD* standard deviation, *CI* confidence interval, *IV* inverse variance

Results of the meta-analysis of Anaerobic: Anaerobic Capacity are presented in Fig. 13. Moderate evidence high-quality anaerobic capacity pooled data from 25 participants indicated no significant changes following operational training (*p* = .56; *g* = 0.11, 95% CI [− 0.26, 0.48]) [26, 30]. The included studies both assessed 300-yard run times.

### Agility

**Fig. 14:**
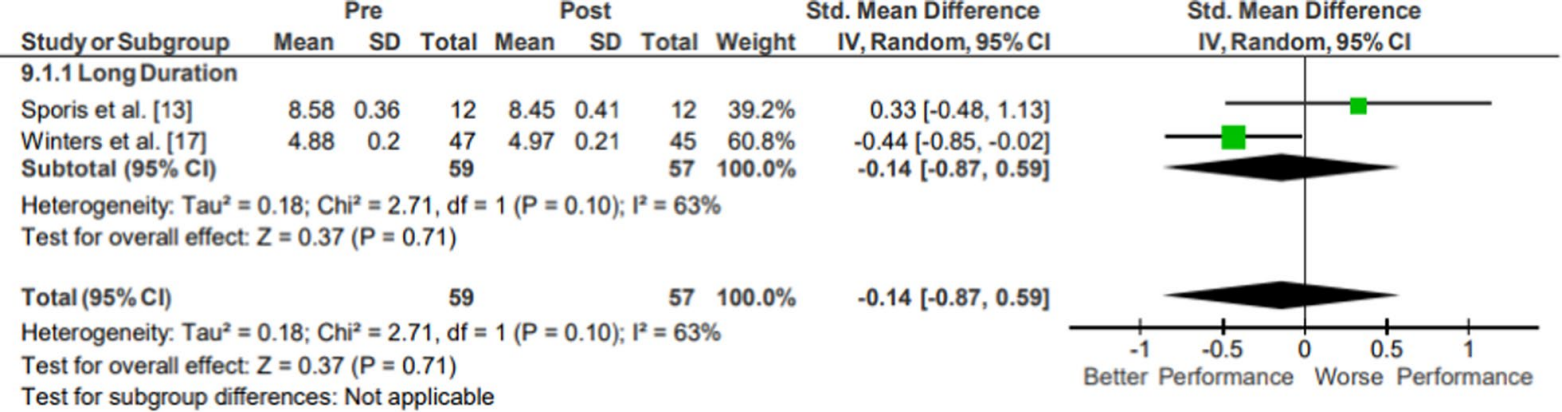
Impact of operational military training on Agility. Abbreviations: *SD* standard deviation, *CI* confidence interval, *IV* inverse variance

Results of the meta-analysis of Agility are presented in Fig. 14. Moderate evidence high-quality agility pooled data from 57 participants indicated no significant changes following operational training (*p* = .71; *g* = − 0.14, 95% CI [− 0.86, 0.59]) [26, 30]. The included studies both assessed agility-style shuttle run times.

## Discussion

This meta-analysis of physical performance across operational military training interventions provides some insight into which constructs of physical performance demonstrate declines in response to operational training. Pooled results indicated limited evidence for large magnitude reductions in Lower Body Power: Jump Power and moderate evidence for small magnitude reductions in Muscular Endurance: Upper Body Endurance and Muscular Endurance: Core Endurance. Short duration results indicated strong evidence for small magnitude reductions in Lower Body Power: Jump Distance and limited evidence for medium magnitude reductions in Lower Body Power: Jump Power. The following constructs were not found to have shown significant improvements or reductions in physical performance: Upper Body Power; Upper, Lower, and Full Body Strength; Aerobic Endurance; Anaerobic Capacity and Power; and Agility. The findings from this meta-analysis highlight the presence of potential temporary reductions in physical performance measures of lower body power and muscular endurance following operational training.

### Potential Contributors to Physical Performance Decline Following Training

There was a small magnitude decline in short-duration jump distance, medium magnitude decline in short-duration jump power, and large magnitude decline in both long and short-duration jump power that was identified by this systematic review. The fatigue of type II muscle fibers may be partially responsible for the decline in power output; jump power and jump distance since these measures rely greatly upon fast-twitch fibers [37]. High-power movements that use type II muscle fibers, such as plyometric jumping activities, require longer recovery times compared to low-power activities that use type I muscle fibers, such as long-distance running [38, 39]. Skeletal muscle adapts structurally and metabolically in response to increased physical activity to allow higher force output and fatigue resistance [40], but only when given sufficient recovery time post-training [41]. In addition to muscle fiber fatigue, central and peripheral neuromuscular fatigue may contribute to the notable decline in lower-extremity power.

Prolonged high-intensity physical activity has been shown to cause a temporary reduction in supplementary motor area excitability [42] and disruptions in extracellular glutamate concentration, affecting voluntary muscular activation [43]. Within the neuromuscular junction, the transmission of neural signals to muscle fibers can be impaired by decrease in acetylcholine, triggered by a frequent high stimulation of neurotransmitter receptors [44]. From an additional physiological perspective, the apoptosis of satellite cells, leading to muscle atrophy and decreased protein synthesis, has been documented in animals subjected to excessive physical activity (11). This adaptation has been projected to occur in military personnel during sustained operational training, displaying as reduced performance in physical metrics during and immediately following said training (11). The intentional restrictions on recovery alongside increases in activity during operational trainings may explain the declines seen in lower body power, implicating this construct as one that may be more impacted by operational training stressors and, by extension, live operations.

This meta-analysis identified moderate evidence of a small magnitude decline in upper and core endurance following operational training. Previous reviews of operational training conditions have determined that increased catabolic hormonal activity in response to physical and mental stress leads to muscle and bone loss in as little as 72 h [11]. In acute conditions, cognitive and physical stress causes increased perceived effort and decreased endurance and force steadiness [45], negatively impacting physiological recovery [46]. Lower recovery rate in response to endurance, strength, and power-based exercise has also been associated with higher musculoskeletal injury rate [43]. Significant declines in lower body power have also been observed during real-time military operations [47], which involve the exact scenarios that simulated operational training strives to replicate. As the declines in lower body power and muscular endurance that occurred in training environments may translate to declines experienced during live operations, military leadership should consider pre-emptively addressing anticipated deficits by continuing to incorporate expected stressors in pre-operation training efforts. Additionally, military leadership may benefit from anticipating and planning around the “worst case scenario” outcomes associated with physical performance declines in their personnel during live operations, as these declines could affect operational success.

Prolonged sleep deprivation coupled with poor sleep quality may have contributed to the reduced Lower Body Power and Muscular Endurance measures after training. While this systematic review did not meta-analyze sleep-related variables, operational stress involving sleep loss and caloric deprivation have been shown to increase fatigue and impair cognition, which reduces physical and task performance [48, 49]. In civilians not subjected to stressful operational training, less than 6 h of sleep significantly reduces next-day power, strength, and endurance performance [50]. Other research from a military-specific population has shown significant declines in aerobic endurance measures of physical performance under conditions that include sleep deprivation [51]. However, as sleep is an uncontrolled confounding variable in many of the included military population studies, the specific influence of sleep deprivation or poor quality of sleep on physical performance is still unclear [51].

### Physical Activity Characteristics Resistant to Change Following Training

The lack of changes found for cardiovascular-type measures, including the Aerobic and Anaerobic constructs, may be explained through acute adaptations to increased physical activity. Increased stress in the form of load carriage can induce cardiovascular adaptations [52], with some evidence suggesting that prolonged physical stress may improve anaerobic and/or aerobic performance [53]. However, muscular stress without adequate recovery time—such as the type of stressors placed upon military personnel during operational training—can attenuate cardiovascular improvements and negate most positive adaptations, especially over periods of several weeks [54]. Thus, the multiple physical demands placed on trained military personnel during operational training may induce no net cardiovascular changes due to both positive and negative adaptation factors.

Of the studies included in the Aerobic construct of this meta-analysis, those that displayed results trending toward improved performance did not measure running times over set distances [8, 30], while those that indicated worsened performance utilized running times as their aerobic measure [17, 26]. This dichotomy between aerobic outcomes for running time measurement versus alternative activities suggests a possible mechanical limitation in aerobic cardiovascular assessment. While we did not analyze lower body endurance due to a lack of clearly specified variables, it is possible that a decline in lower body endurance due to similar factors driving the declines in upper and core endurance may be responsible for the decrease in aerobic performance in the three studies assessing running time [17, 19, 26]. If the muscles involved in running experienced fatigue, it is possible that the limiting factor in running time could be fatigue affecting lower body endurance instead of aerobic capability [55]. This explanation would align the findings of no net change in Aerobic Endurance more closely with evidence that shows increases in cardiovascular capacity after time spent under physically stressful conditions [52, 53].

The studies included under the Anaerobic construct demonstrated high methodological heterogeneity. Conflicting results in the Anaerobic Power and Capacity subcategories suggest potential influence of intervention factors that were not accounted for in this meta-analysis. Because energy systems and neuromuscular activation patterns are often similar between anaerobic and power-type exercises [56], it was anticipated that adaptations in anaerobic performance would be similar to those seen for lower body power performance. However, these expected results were not found in this meta-analysis. Two studies that examined both jump distance and anaerobic events revealed improvements in both variables at post-intervention [26, 30], indicating potential decline-mitigating factors within these studies that were not analyzed for this review. While speculative, previous research has shown that muscular adaptation to imposed demand occurs with prolonged strength training [57], adaptations that may be responsible for improved fast-twitch muscular response despite facing other stressful training demands. As several of the included anaerobic power and capacity studies consisted of long-term intervention durations, it is plausible that the participants in these studies developed fast-twitch muscle fiber adaptations over time, mitigating the expected negative effects of operational training on anaerobic performance.

Similar to the findings for anaerobic capacity and power, the results for lower, upper, and full body strength in this meta-analysis contradicted expectations. Research showed significant reductions in lower extremity strength after conducting running activities, especially anaerobic-based high-intensity interval trainings similar to the activities included in operational training interventions [58]. In each respective analysis for lower body strength, upper body strength, and full body strength, methodological differences in exercises utilized across each category may have contributed to the findings of a lack of changes in strength. The Full Body Strength construct results trended toward decline, but not significantly, and both subcategories of Upper Body Strength displayed mixed results. The body enters a catabolic state under high stress conditions that involve sleep deprivation [59] or which are rated as causing higher perceived stress [60]. In this state, metabolic processes break down muscle proteins, resulting in muscle wasting and decreased strength regardless of the concurrent presence of caloric restriction [61]. Like the declines in power production from prolonged physical stress with lack of recovery, declines in strength would have been anticipated post-training.

### Limitations

The results of our meta-analysis should be interpreted in the context of several limitations, with the most influential being methodological heterogeneity of included studies. Existing research on operational training frequently focuses on constructs other than physical performance, such as physiological (e.g. circulating hormone levels) and cognitive (e.g. memory-challenging tasks) assessments [6, 11]. For studies that do evaluate physical performance during operational training, the military training nomenclature varies. Some trainings are specified, such as with “Navy Survival, Evasion, Resistance, and Escape (SERE)” training or “Special Forces Training Course”, while others are labelled more generally, such as “field training” or “sustained operation training”, affecting intervention searchability. This variability in operational training also presents its own limitation, in that adaptations occurring throughout different types of training may not be comparable due to the vast differences in military training modalities. Finally, one of the largest limitations in the applicability of this review stems from the limited number of studies included in some of the constructs or subcategories, with a few analyses conducted on only two sets of data. More robust analyses, containing data from several interventions of highly similar nature, would be desirable for several of the constructs included in this meta-analysis.

### Recommendations and Future Considerations

Alongside primary outcome measures like physiological or cognitive effects of operational training, future research should consider incorporating deliberate measurement of physical performance to allow for more holistic conclusions. While physical performance testing in military training settings has traditionally focused on provision of “field expedient” measures, experts within the military research community have recently advised moving toward better standardization of physical testing [2]. Such standardization could develop from the application of existing standardized fitness testing, such as the recently adopted Army Combat Fitness Test (ACFT). Administration of some or all ACFT events during operational training would provide better physical testing standardization while also providing comparative data of physical performance in an applied, operationally stressed environment versus a controlled, garrison-style environment. Finally, while this meta-analysis provides information on the acute post-intervention effects of operational training, future research could expand on these findings by assessing the post-intervention effects at multiple time points after the trainings’ conclusions, giving better insight into the impact of a recovery period.

From the perspective of military leadership, the findings of this meta-analysis suggest that military personnel experience a decrease in measures of both power and endurance immediately after the conclusion of a high stress simulated operational training. These findings reflect both short-term and long-term training experiences, meaning all trainings of this nature may reduce physical performance capabilities, at least for the lower body power and upper/core endurance activities. While the exact permanence of these physical performance decrements was not analyzed, it is likely that most of the observed reductions are temporary, as some of the included research provided evidence supporting a return of physical performance following the examined training [30].

In a state of physical exhaustion due to high-stress environments, military personnel are at a heightened risk of injury and are not able to complete operational tasks to the same level of effectiveness as they could while replenished [11]. Regular participation in operational training that simulates the stresses experienced during real operations may still be the best way to prepare military personnel for the extreme physical conditions faced in combat or similar situations. However, by ensuring personnel are given adequate recovery time post-training and by ensuring that power and endurance fitness activities are routinely incorporated into regular physical training programs, military leaders may reduce injury risk and diminishment of task capabilities while still providing preparatory operational experience.

In real operational circumstances, adequate physical recovery may not always be feasible. Leadership can still mediate declining performance and increased injury risk through planning for provision of other aspects of recovery, as multiple sources of extreme stress may heighten the rate of performance deterioration. For example, if good quality sleep is not achievable in an operational task, adequate energy provision to meet nutritional needs may offset some of the negative physical effects brought on by sleep deprivation. As most studies of operational training or live operations assess multiple stressors at a time [48], future research would benefit from analysis of the independent and separate effects of sleep deprivation, caloric restriction, and other sources of potential distress.

## Conclusions

This analysis of 17 studies that examined the impact of simulated military operational training events on measures of physical performance revealed small declines in measures of Lower Body Power: Jump Distance—short duration (strong evidence), Upper Body Endurance (moderate evidence), and Core Endurance (moderate evidence). This analysis found a large decline in Lower Body Power: Jump Power; however, this finding was supported by limited evidence. Although no declines were identified in the collective analysis of other physical performance measures, it is nonetheless likely that most operational training events are associated with frequent, likely acute reductions in several constructs that affect overall physical performance.

## Supplementary Information

Additional file 1.

Additional file 2.

Additional file 3.

Additional file 4.

Additional file 5.

## Data Availability

The datasets used and/or analysed during the current study are available from the corresponding author on reasonable request.

## Abbreviations

SMD: Standardized mean differences
PRISMA: Preferred reporting items for systematic reviews and meta-analysis
NIH: National Institutes of Health
ACFT: Army combat fitness test
SERE: Survival, evasion, resistance, and escape

## Declarations

### Disclaimer

JJF is an employee of the U.S. Government and this work was prepared as part of his official duties. Title 17, U.S.C. §105 provides that copyright protection under this title is not available for any work of the U.S. Government. Title 17, U.S.C. §101 defines a U.S. Government work as work prepared by a military service member or employee of the U.S. Government as part of that person’s official duties. The views expressed are those of the authors and do not necessarily reflect the official policy or position of Uniformed Service University, the Department of the Navy, the Department of Defense, the Department of Veterans Affairs, nor the U.S. Government.

## References

[1] Miller NL, Shattuck LG, Matsangas P. Sleep and fatigue issues in continuous operations: a survey of US Army officers. Behav Sleep Med. 2011;9(1):53–65. 10.1080/15402002.2011.533994. 21218294 10.1080/15402002.2011.533994

[2] Nindl BC, Billing DC, Drain JR, et al. Perspectives on resilience for military readiness and preparedness: report of an international military physiology roundtable. J Sci Med Sport. 2018;21(11):1116–24. 10.1016/j.jsams.2018.05.005. 29886134 10.1016/j.jsams.2018.05.005

[3] Conkright WR, Barringer ND, Lescure PB, Feeney KA, Smith MA, Nindl BC. Differential recovery rates of fitness following U.S. Army Ranger training. J Sci Med Sport. 2020;23(5):529–34. 10.1016/j.jsams.2019.12.010. 31870679 10.1016/j.jsams.2019.12.010

[4] Robson, Sean and Thomas Manacapilli, Enhancing Performance Under Stress: Stress Inoculation Training for Battlefield Airmen. Santa Monica, CA: RAND Corporation, 2014. https://www.rand.org/pubs/research_reports/RR750.html. Also available in print form.

[5] Haslam DE. Sustained operations and military performance. Behav Res Meth Instrum Comput. 1985;17:90–5. 10.3758/BF03200901.

[6] Vrijkotte S, Roelands B, Meeusen R, Pattyn N. Sustained military operations and cognitive performance. Aerosp Med Hum Perform. 2016;87(8):718–27. 10.3357/AMHP.4468.2016. 27634607 10.3357/AMHP.4468.2016

[7] Greer J. Training: The foundation for success in combat. Heritage Foundation. 2018;4. Accessed January 31, 2023. https://www.heritage.org/military-strength-topical-essays/2019-essays/training-the-foundation-success-combat#.

[8] Varanoske AN, Wells AJ, Kozlowski GJ, et al. Effects of β-alanine supplementation on physical performance, cognition, endocrine function, and inflammation during a 24 h simulated military operation. Physiol Rep. 2018;6(24): e13938. 10.14814/phy2.13938. 30565426 10.14814/phy2.13938 PMC6299243

[9] Ojanen T, Häkkinen K, Vasankari T, Kyröläinen H. Changes in physical performance during 21 d of military field training in warfighters. Mil Med. 2018;183(5–6):e174–81. 10.1093/milmed/usx049. 29420780 10.1093/milmed/usx049

[10] Delahaij R, Gaillard AWK, Soeters JMLM. Stress Training and the New Military Environment. In Human Dimensions in Military Operations – Military Leaders’ Strategies for Addressing Stress and Psychological Support (pp. 17A-1–17A-10). Meeting Proceedings RTO-MP-HFM-134, Paper 17A. Neuilly-sur-Seine, France: RTO. (2006) Available from: http://www.rto.nato.int/abstracts.asp.

[11] Henning PC, Park BS, Kim JS. Physiological decrements during sustained military operational stress. Mil Med. 2011;176(9):991–7. 10.7205/milmed-d-11-00053. 21987955 10.7205/milmed-d-11-00053

[12] Heilbronn B, Doma K, Sinclair W, Connor J, Irvine-Brown L, Leicht A. Acute fatigue responses to occupational training in military personnel: a systematic review and meta-analysis. Mil Med. 2022. 10.1093/milmed/usac144. 35639912 10.1093/milmed/usac144 PMC10187475

[13] Silva FB, Vaisman M, Ponce T, et al. A systematic review of hormone levels, biomarkers of cellular injury and oxidative stress in multi-stressor military field training exercises. Arch Endocrinol Metab. 2022. 10.20945/2359-3997000000443. 35289515 10.20945/2359-3997000000443 PMC9832854

[14] Beckner ME, Conkright WR, Eagle SR, et al. Impact of simulated military operational stress on executive function relative to trait resilience, aerobic fitness, and neuroendocrine biomarkers. Physiol Behav. 2021;236: 113413. 10.1016/j.physbeh.2021.113413. 33811909 10.1016/j.physbeh.2021.113413

[15] Chester AL, Edwards AM, Crowe M, Quirk F. Physiological, biochemical, and psychological responses to environmental survival training in the Royal Australian Air Force. Mil Med. 2013;178(7):e829–35. 10.7205/MILMED-D-12-00499. 23820360 10.7205/MILMED-D-12-00499

[16] Gan LSH, Fan PWP, Zhang J, et al. Changes in energy balance, body composition, metabolic profile and physical performance in a 62-day Army Ranger training in a hot-humid environment. J Sci Med Sport. 2022;25(1):89–94. 10.1016/j.jsams.2021.08.005. 34507882 10.1016/j.jsams.2021.08.005

[17] Hoffman JR, Landau G, Stout JR, et al. β-alanine supplementation improves tactical performance but not cognitive function in combat soldiers. J Int Soc Sports Nutr. 2014;11(1):15. 10.1186/1550-2783-11-15. 24716994 10.1186/1550-2783-11-15 PMC3983672

[18] Margolis LM, Murphy NE, Martini S, et al. Effects of winter military training on energy balance, whole-body protein balance, muscle damage, soreness, and physical performance. Appl Physiol Nutr Metab. 2014;39(12):1395–401. 10.1139/apnm-2014-0212. 25386980 10.1139/apnm-2014-0212

[19] Montain SJ, Shippee RL, Tharion WJ. Carbohydrate-electrolyte solution effects on physical performance of military tasks. Aviat Space Environ Med. 1997;68(5):384–91. 9143747

[20] Nindl BC, Leone CD, Tharion WJ, et al. Physical performance responses during 72 h of military operational stress. Med Sci Sports Exerc. 2002;34(11):1814–22. 10.1097/00005768-200211000-00019. 12439088 10.1097/00005768-200211000-00019

[21] Nindl BC, Barnes BR, Alemany JA, Frykman PN, Shippee RL, Friedl KE. Physiological consequences of US Army Ranger training. Med Sci Sports Exerc. 2007;39(8):1380–7. 10.1249/MSS.0b013e318067e2f7. 17762372 10.1249/MSS.0b013e318067e2f7

[22] Ojanen T, Kyröläinen H, Igendia M, Häkkinen K. Effect of prolonged military field training on neuromuscular and hormonal responses and shooting performance in warfighters. Mil Med. 2018;183(11–12):e705–12. 10.1093/milmed/usy122. 29860348 10.1093/milmed/usy122

[23] Ritland BM, Naylor JA, Bessey AF, et al. Transitioning from daytime to nighttime operations in military training has a temporary negative impact on dynamic balance and jump performance in US Army Rangers. J Sci Med Sport. 2021;24(9):919–24. 10.1016/j.jsams.2021.02.013. 33750655 10.1016/j.jsams.2021.02.013

[24] Różański P, Jówko E, Tomczak A. Assessment of the levels of oxidative stress, muscle damage, and psychomotor abilities of special force soldiers during military survival training. Int J Environ Res Public Health. 2020;17(13):4886. 10.3390/ijerph17134886. 32645886 10.3390/ijerph17134886 PMC7370038

[25] Salonen M, Huovinen J, Kyröläinen H, Piirainen JM, Vaara JP. Neuromuscular performance and hormonal profile during military training and subsequent recovery period. Mil Med. 2019;184(3–4):e113–9. 10.1093/milmed/usy176. 30053107 10.1093/milmed/usy176

[26] Sporiš G, Harasin D, Bok D, Matika D, Vuleta D. Effects of a training program for special operations battalion on soldiers’ fitness characteristics. J Strength Cond Res. 2012;26(10):2872–82. 10.1519/JSC.0b013e318242966c. 22130399 10.1519/JSC.0b013e318242966c

[27] Szivak TK, Lee EC, Saenz C, Flanagan SD, Focht BC, Volek JS, Maresh CM, Kraemer WJ. Adrenal stress and physical performance during military survival training. Aerosp Med Hum Perform. 2018;89(2):99–107. 29463354 10.3357/AMHP.4831.2018

[28] Tomczak A. Coordination Motor Skills of Military Pilots Subjected to Survival Training. J Strength Cond Res. 2015;29(9):2460–4. 10.1519/JSC.0000000000000910. 25719921 10.1519/JSC.0000000000000910

[29] Welsh TT, Alemany JA, Montain SJ, et al. Effects of intensified military field training on jumping performance. Int J Sports Med. 2008;29(1):45–52. 10.1055/s-2007-964970. 17879876 10.1055/s-2007-964970

[30] Winters JD, Heebner NR, Johnson AK, et al. Altered physical performance following advanced special operations tactical training. J Strength Cond Res. 2021;35(7):1809–16. 10.1519/JSC.0000000000003087. 30985522 10.1519/JSC.0000000000003087

[31] Moher D, Liberati A, Tetzlaff J, Altman DG, PRISMA Group. Preferred reporting items for systematic reviews and meta-analyses: the PRISMA statement. PLoS Med. 2009;6(7): e1000097. 10.1371/journal.pmed.1000097. 19621072 10.1371/journal.pmed.1000097 PMC2707599

[32] Keramidas ME, Gadefors M, Nilsson LO, Eiken O. Physiological and psychological determinants of whole-body endurance exercise following short-term sustained operations with partial sleep deprivation. Eur J Appl Physiol. 2018;118(7):1373–84. 10.1007/s00421-018-3869-0. 29687266 10.1007/s00421-018-3869-0 PMC6028900

[33] Conkright WR, Beckner ME, Sinnott AM, et al. Neuromuscular performance and hormonal responses to military operational stress in men and women. J Strength Cond Res. 2021;35(5):1296–305. 10.1519/JSC.0000000000004013. 33780395 10.1519/JSC.0000000000004013

[34] Haff, GG, Triplett, TN. Essentials of strength training and conditioning. National Strength & Conditioning Association (U.S.), issuing body. 4th ed. Human Kinetics; 2016.

[35] Higgins JPT, Thomas J, Chandler J, et al. (editors). Cochrane Handbook for Systematic Reviews of Interventions version 6.3 (updated February 2022). Cochrane, 2022.

[36] van Tulder M, Furlan A, Bombardier C, et al. Updated method guidelines for systematic reviews in the cochrane collaboration back review group. Spine (Phila Pa 1976). 2003 Jun 15;28(12):1290–9. 10.1097/01.BRS.0000065484.95996.AF 12811274

[37] Wakeling JM, Uehli K, Rozitis AI. Muscle fibre recruitment can respond to the mechanics of the muscle contraction. J R Soc Interface. 2006;3(9):533–44. 10.1098/rsif.2006.0113. 16849250 10.1098/rsif.2006.0113 PMC1664648

[38] Macaluso F, Isaacs AW, Myburgh KH. Preferential type II muscle fiber damage from plyometric exercise. J Athl Train. 2012;47(4):414–20. 10.4085/1062-6050-47.4.13. 22889657 10.4085/1062-6050-47.4.13 PMC3396301

[39] Lievens E, Klass M, Bex T, Derave W. Muscle fiber typology substantially influences time to recover from high-intensity exercise. J Appl Physiol. 2020;128(3):648–59. 10.1152/japplphysiol.00636.2019. 31999527 10.1152/japplphysiol.00636.2019

[40] Bogdanis GC. Effects of physical activity and inactivity on muscle fatigue. Front Physiol. 2012;3:142. 10.3389/fphys.2012.00142. 22629249 10.3389/fphys.2012.00142 PMC3355468

[41] Pearson AM. Muscle growth and exercise. Crit Rev Food Sci Nutr. 1990;29(3):167–96. 10.1080/10408399009527522. 2222798 10.1080/10408399009527522

[42] Coco M, Perciavalle V, Cavallari P, Perciavalle V. Effects of an exhaustive exercise on motor skill learning and on the excitability of primary motor cortex and supplementary motor area. Medicine. 2016;95(11): e2978. 10.1097/MD.0000000000002978. 26986109 10.1097/MD.0000000000002978 PMC4839890

[43] Tornero-Aguilera JF, Jimenez-Morcillo J, Rubio-Zarapuz A, Clemente-Suárez VJ. Central and peripheral fatigue in physical exercise explained: a narrative review. Int J Environ Res Public Health. 2022;19(7):3909. 10.3390/ijerph19073909. 35409591 10.3390/ijerph19073909 PMC8997532

[44] Sam C, Bordoni B. Physiology, Acetylcholine. [Updated 2023 Apr 10]. In: StatPearls [Internet]. Treasure Island (FL): StatPearls Publishing; 2024 Jan-. Available from: https://www.ncbi.nlm.nih.gov/books/NBK557825/). 32491757

[45] Tyagi O, Mehta R. A Methodological Framework to Capture Neuromuscular Fatigue Mechanisms Under Stress. Frontiers in Neuroergonomics. 2021. 10.3389/fnrgo.2021.779069. 38235237 10.3389/fnrgo.2021.779069 PMC10790877

[46] Mehta RK, Agnew MJ. Influence of mental workload on muscle endurance, fatigue, and recovery during intermittent static work. Eur J Appl Physiol. 2012;112(8):2891–902. 10.1007/s00421-011-2264-x. 22143842 10.1007/s00421-011-2264-x

[47] Murphy NE, Carrigan CT, Philip Karl J, Pasiakos SM, Margolis LM. Threshold of energy deficit and lower-body performance declines in military personnel: a meta-regression. Sports Med. 2018;48(9):2169–78. 10.1007/s40279-018-0945-x. 29949108 10.1007/s40279-018-0945-x

[48] Passi T, Lukander K, Laarni J, et al. Effects of overnight military training and acute battle stress on the cognitive performance of soldiers in simulated urban combat. Front Psychol. 2022;13: 925157. 10.3389/fpsyg.2022.925157. 35959037 10.3389/fpsyg.2022.925157 PMC9360769

[49] Beckner ME, Lieberman HR, Hatch-McChesney A, et al. Effects of energy balance on cognitive performance, risk-taking, ambulatory vigilance and mood during simulated military sustained operations (SUSOPS). Physiol Behav. 2023;258: 114010. 10.1016/j.physbeh.2022.114010. 36349660 10.1016/j.physbeh.2022.114010

[50] Craven J, McCartney D, Desbrow B, et al. Effects of acute sleep loss on physical performance: a systematic and meta-analytical review. Sports Med. 2022;52(11):2669–90. 10.1007/s40279-022-01706-y. 35708888 10.1007/s40279-022-01706-y PMC9584849

[51] Grandou C, Wallace L, Fullagar HHK, Duffield R, Burley S. The effects of sleep loss on military physical performance. Sports Med. 2019;49(8):1159–72. 10.1007/s40279-019-01123-8. 31102110 10.1007/s40279-019-01123-8

[52] Miller JF, Stamford BA. Intensity and energy cost of weighted walking vs. running for men and women. J Appl Physiol. 1987;62(4):1497–501. 10.1152/jappl.1987.62.4.1497. 3597221 10.1152/jappl.1987.62.4.1497

[53] Thomas M, Pohl MB, Shapiro R, Keeler J, Abel MG. Effect of load carriage on tactical performance in special weapons and tactics operators. J Strength Cond Res. 2018;32(2):554–64. 10.1519/JSC.0000000000002323. 29120978 10.1519/JSC.0000000000002323

[54] Santtila M, Häkkinen K, Karavirta L, Kyröläinen H. Changes in cardiovascular performance during an 8-week military basic training period combined with added endurance or strength training. Mil Med. 2016;181(9):1165. 10.7205/MILMED-D-16-0026648. 27612372 10.7205/MILMED-D-16-00266

[55] Wan JJ, Qin Z, Wang PY, Sun Y, Liu X. Muscle fatigue: general understanding and treatment. Exp Mol Med. 2017;49(10): e384. 10.1038/emm.2017.194. 28983090 10.1038/emm.2017.194 PMC5668469

[56] Zierath JR, Hawley JA. Skeletal muscle fiber type: influence on contractile and metabolic properties. PLoS Biol. 2004;2(10): e348. 10.1371/journal.pbio.0020348. 15486583 10.1371/journal.pbio.0020348 PMC521732

[57] Hughes DC, Ellefsen S, Baar K. Adaptations to endurance and strength training. Cold Spring Harb Perspect Med. 2018;8(6): a029769. 10.1101/cshperspect.a029769. 28490537 10.1101/cshperspect.a029769 PMC5983157

[58] Riazati S, Caplan N, Matabuena M, Hayes PR. Fatigue induced changes in muscle strength and gait following two different intensity, energy expenditure matched runs. Front Bioeng Biotechnol. 2020;8:360. 10.3389/fbioe.2020.00360. 32391353 10.3389/fbioe.2020.00360 PMC7188949

[59] Lamon S, Morabito A, Arentson-Lantz E, et al. The effect of acute sleep deprivation on skeletal muscle protein synthesis and the hormonal environment. Physiol Rep. 2021;9(1): e14660. 10.14814/phy2.14660. 33400856 10.14814/phy2.14660 PMC7785053

[60] Poornima KN, Karthick N, Sitalakshmi R. Study of the effect of stress on skeletal muscle function in geriatrics. J Clin Diagn Res. 2014;8(1):8–9. 10.7860/JCDR/2014/7014.3966. 24596710 10.7860/JCDR/2014/7014.3966 PMC3939594

[61] Hasselgren PO. Catabolic response to stress and injury: implications for regulation. World J Surg. 2000;24(12):1452–9. 10.1007/s002680010262. 11193708 10.1007/s002680010262

