# Supplementary material for: Changes in Physical Performance Following Operational Military Training: A Meta-Analysis
Source: Sports Med Open. 2025 Feb 13;11:16. doi: 10.1186/s40798-025-00815-y (PMC11825424; doi:10.1186/s40798-025-00815-y)

Supplemental Electronic Material – Figure 1

Methodological Quality Assessment – Study Rankings


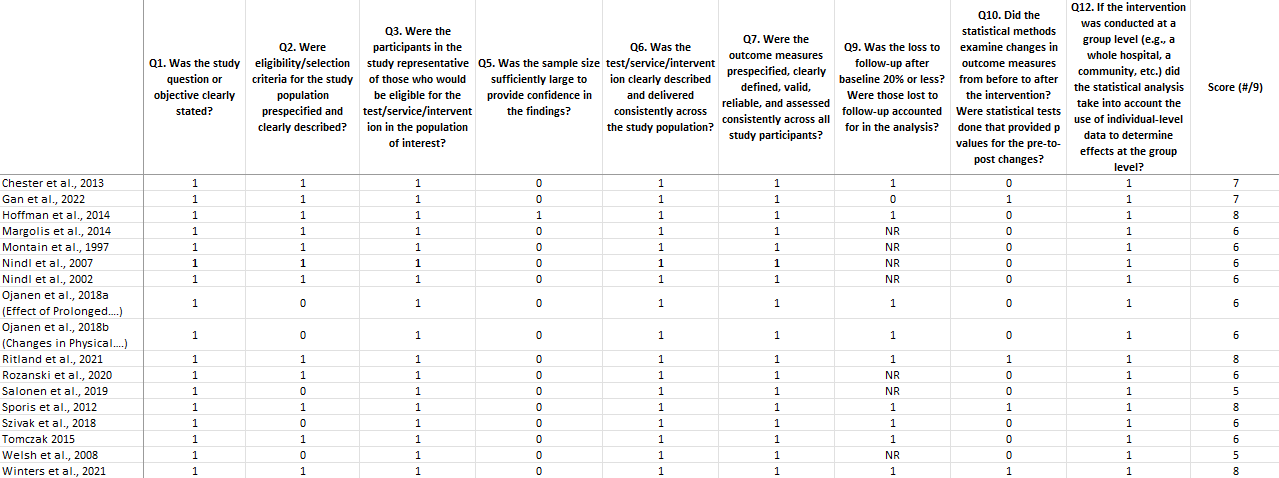

Supplement: Supplementary file 1 — Additional file 1. [file 40798_2025_815_MOESM1_ESM.docx]
