# Supplementary material for: Changes in Physical Performance Following Operational Military Training: A Meta-Analysis
Source: Sports Med Open. 2025 Feb 13;11:16. doi: 10.1186/s40798-025-00815-y (PMC11825424; doi:10.1186/s40798-025-00815-y)

Supplemental Electronic Material – Figures 2-4

Funnel Plots – Significant Categories

Fig 2. Lower Body Power: Distance


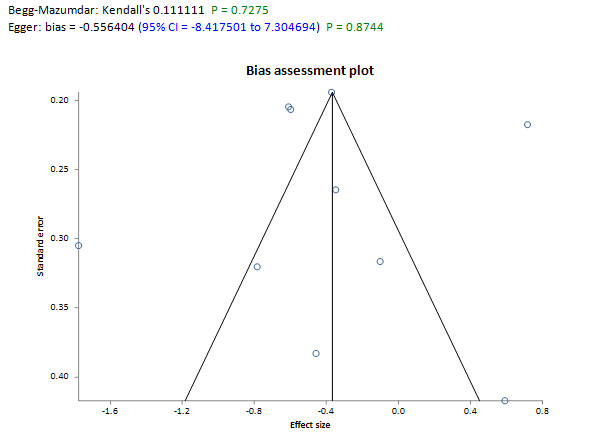


Fig 3. Lower Body Power: Power


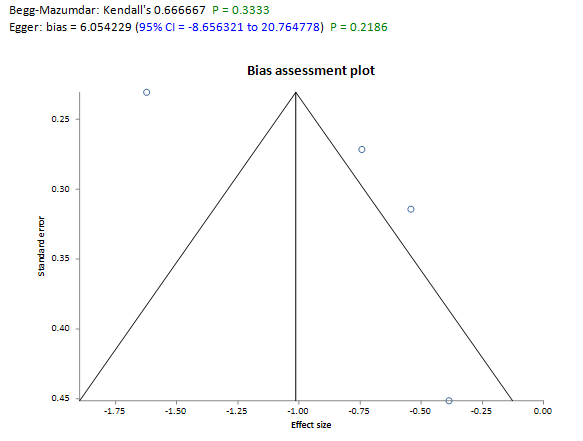


Fig 4. Muscular Endurance: Upper Endurance


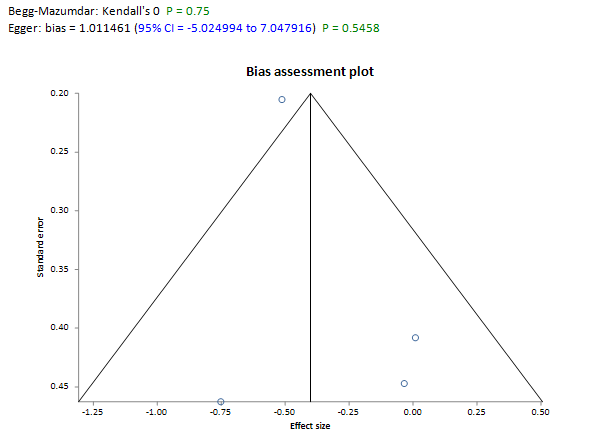

Supplement: Supplementary file 2 — Additional file 2. [file 40798_2025_815_MOESM2_ESM.docx]
