# Supplementary material for: Changes in Physical Performance Following Operational Military Training: A Meta-Analysis
Source: Sports Med Open. 2025 Feb 13;11:16. doi: 10.1186/s40798-025-00815-y (PMC11825424; doi:10.1186/s40798-025-00815-y)
Supplement: Supplementary file 3 — Additional file 3. [file 40798_2025_815_MOESM3_ESM.docx]

**Appendix 1:**

**Database Search Configurations**

| **Database** | **Basic Search Term** | **Date(s) Searched** |
| --- | --- | --- |
| PubMed | military stress physical performance | 01/30/2022  03/19/2022 |
| PubMed | military sustained operations physical fitness performance | 2/1/2022  3/19/2022 |
| PubMed | military operational training physical performance | 01/30/2022  03/20/2022 |
| PubMed | military prolonged operational training physical performance | 2/1/2022  3/20/2022 |
| PubMed | military field training physical performance | 2/1/2022  3/19/2022 |
| DTIC: PubDefense | military operational training physical activity changes | 1/31/2021 |
| DTIC: PubDefense | military training physical performance | 1/31/2021 |
| DTIC: PubDefense | operational training physical performance | 1/31/2021 |
| Proquest | military physical fitness performance operations field training | 2/2/2022 |
| Proquest | military physical fitness sustained operations training | 2/2/2022 |
| Proquest | military prolonged operational training physical performance | 2/2/2022 |
| Embase | military operational training physical performance | 2/5/2022 |
| Embase | military prolonged training physical performance | 2/5/2022 |
| Embase | military field training physical performance | 2/5/2022 |
| Embase | military stressful training physical changes | 2/5/2022 |
| Web of Science | military operational training physical performance | 2/6/2022 |
| Web of Science | military prolonged training physical performance | 2/6/2022 |
| Web of Science | military field training physical performance | 2/6/2022 |
| Web of Science | military stressful training physical changes | 2/6/2022 |
| Web of Science | military survival training physical performance | 2/6/2022 |
| CINAHL Plus with Full Text | military operational training physical performance | 2/11/2022 |
| CINAHL Plus with Full Text | military prolonged training physical performance | 2/11/2022 |
| CINAHL Plus with Full Text | military field training physical performance | 2/11/2022 |
| CINAHL Plus with Full Text | military stressful training physical changes | 2/11/2022 |
| CINAHL Plus with Full Text | military survival training physical performance | 2/11/2022 |
