# Supplementary material for: Changes in Physical Performance Following Operational Military Training: A Meta-Analysis
Source: Sports Med Open. 2025 Feb 13;11:16. doi: 10.1186/s40798-025-00815-y (PMC11825424; doi:10.1186/s40798-025-00815-y)
Supplement: Supplementary file 4 — Additional file 4. [file 40798_2025_815_MOESM4_ESM.docx]

**Appendix 2:**

**National Institutes of Health (NIH) Quality Assessment Tool for Before-After (Pre-Post)**

**Studies With No Control Group**

All questions answered per each evaluated study with “yes” (1 point), “no” (0 point), “cannot determine” (0 point), “not applicable” (0 point), or “not reported” (0 point).

Questions 4, 8, and 11 were excluded from the criteria used for this meta-analysis due to lack of applicability to the types of studies included in the analysis (e.g. question 8, “Were the people assessing the outcomes blinded to the participants' exposures/interventions?”, was not necessary for the observational studies conducted).

Q1. Was the study question or objective clearly stated?

Q2. Were eligibility/selection criteria for the study population prespecified and clearly described?

Q3. Were the participants in the study representative of those who would be eligible for the test/service/intervention in the population of interest?

Q4. Were all eligible participants that met the prespecified entry criteria enrolled?

Q5. Was the sample size sufficiently large to provide confidence in the findings?

Q6. Was the test/service/intervention clearly described and delivered consistently across the study population?

Q7. Were the outcome measures prespecified, clearly defined, valid, reliable, and assessed consistently across all study participants?

Q8. Were the people assessing the outcomes blinded to the participants' exposures/interventions?

Q9. Was the loss to follow-up after baseline 20% or less? Were those lost to follow-up accounted for in the analysis?

Q10. Did the statistical methods examine changes in outcome measures from before to after the intervention? Were statistical tests done that provided p values for the pre-to-post changes?

Q11. Were outcome measures of interest taken multiple times before the intervention and multiple times after the intervention (i.e., did they use an interrupted time-series design)?

Q12. If the intervention was conducted at a group level (e.g., a whole hospital, a community, etc.) did the statistical analysis take into account the use of individual-level data to determine effects at the group level?
