# Supplementary material for: Changes in Physical Performance Following Operational Military Training: A Meta-Analysis
Source: Sports Med Open. 2025 Feb 13;11:16. doi: 10.1186/s40798-025-00815-y (PMC11825424; doi:10.1186/s40798-025-00815-y)
Supplement: Supplementary file 5 — Additional file 5. [file 40798_2025_815_MOESM5_ESM.docx]

**Appendix 3:**

**Modified Van Tulder Levels of Evidence Criteria**

***strong evidence***

1. Statistically homogeneous (I^2^<50%)
2. 3+ studies
3. 2+ HQ studies

***moderate evidence***

1. Statistically heterogeneous (I^2^>50%)
2. 2+ studies
3. 1+ HQ study

OR

1. 2+ studies
2. MQ or LQ studies
3. statistically homogeneous (I^2^<50%)

***limited evidence***

1. 1 HQ study, or
2. 2+ MQ or LQ studies AND statistically heterogeneous (I^2^>50%)

***Very limited evidence***

1. 1 MQ or LQ study
